# Impact of Crystal Size and Morphology on Switchability Characteristics in Pillared-Layer Metal-Organic Framework DUT-8(Ni)

**DOI:** 10.3389/fchem.2021.674566

**Published:** 2021-05-13

**Authors:** Leila Abylgazina, Irena Senkovska, Richard Engemann, Sebastian Ehrling, Tatiana E. Gorelik, Negar Kavoosi, Ute Kaiser, Stefan Kaskel

**Affiliations:** ^1^Institute of Inorganic Chemistry I, Technische Universität Dresden, Dresden, Germany; ^2^3P Instruments, Odelzhausen, Germany; ^3^Electron Microscopy Group of Materials Science (EMMS), Central Facility for Electron Microscopy, Ulm University, Ulm, Germany; ^4^Landeslabor Berlin-Brandenburg, Frankfurt, Germany

**Keywords:** crystal habit, switchable MOFs, crystal size, crystal morphology, gate pressure MOF, pillared-layer MOFs

## Abstract

Variation of the crystallite size in flexible porous coordination polymers can significantly influence or even drastically change the flexibility characteristics. The impact of crystal morphology, however, on the dynamic properties of flexible metal-organic frameworks (MOFs) is poorly investigated so far. In the present work, we systematically modulated the particle size of a model gate pressure MOF (DUT-8(Ni), Ni_2_(2,6-ndc)_2_(dabco), 2,6-ndc−2,6-naphthalenedicarboxylate, dabco−1,4-diazabicyclo[2.2.2]octane) and investigated the influence of the aspect ratio, length, and width of anisotropically shaped crystals on the gate opening characteristics. DUT-8 is a member of the pillared-layer MOF family, showing reversible structural transition, i.e., upon nitrogen physisorption at 77 K. The framework crystalizes as rod-like shaped crystals in conventional synthesis. To understand which particular crystal surfaces dominate the phenomena observed, crystals similar in size and differing in morphology were involved in a systematic study. The analysis of the data shows that the width of the rods (corresponding to the crystallographic directions along the layer) represents a critical parameter governing the dynamic properties upon adsorption of nitrogen at 77 K. This observation is related to the anisotropy of the channel-like pore system and the nucleation mechanism of the solid-solid phase transition triggered by gas adsorption.

**Graphical Abstract:**
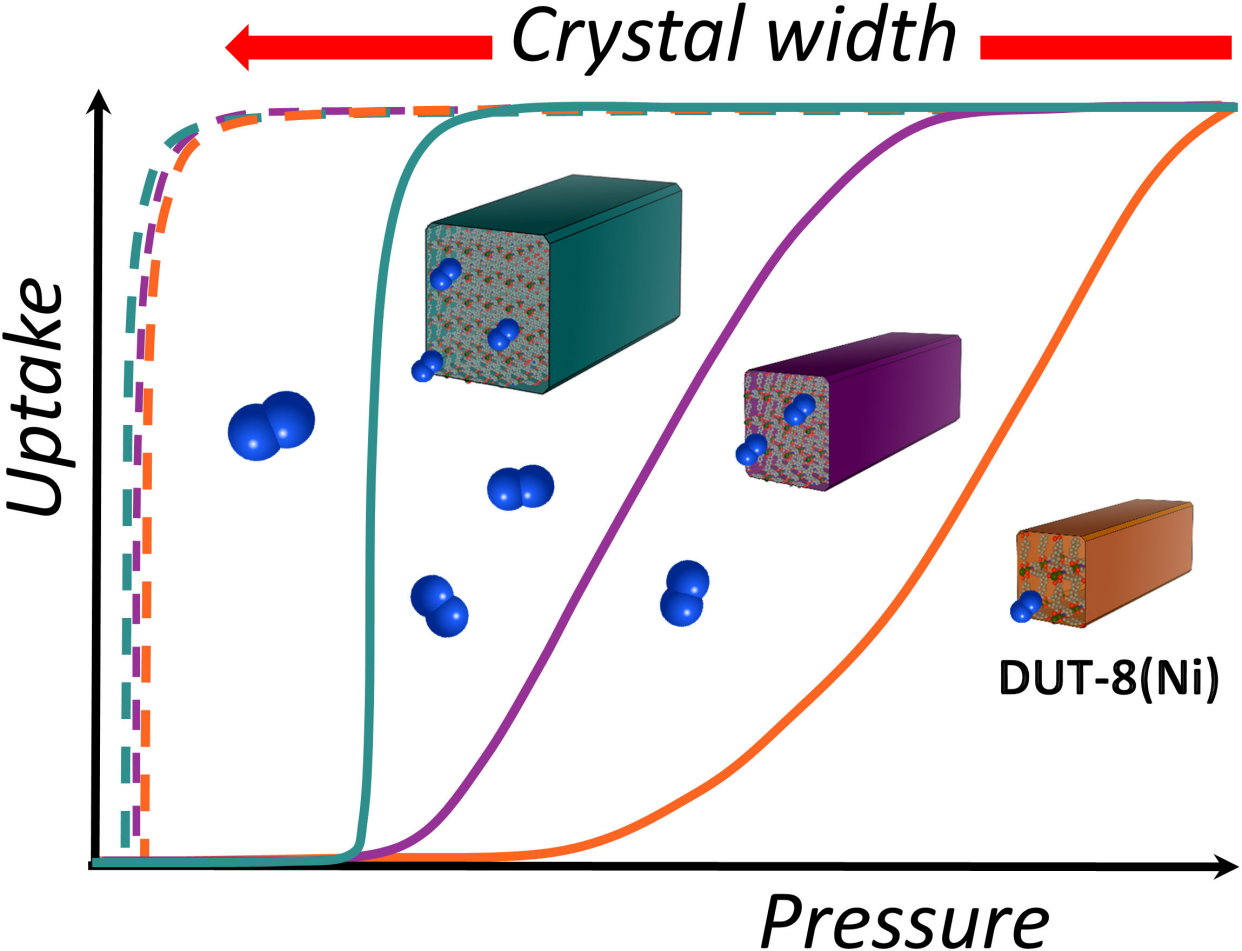
Particle size dependent adsorption behavior of DUT-8(Ni).

## Introduction

Metal-organic frameworks (MOFs) are nowadays well-known as a class of porous coordination polymers which are constructed from organic linkers and metal clusters by modular building principle (Schneemann et al., 2014; Elsaidi et al., 2018). The so-called “third generation” of MOFs represents a subset of these materials, showing reversible stimuli-responsive structural transitions upon adsorption/desorption of fluids, temperature or pressure change (Boutin et al., 2013; Gagnon et al., 2013; Wieme et al., 2018; Collings and Goodwin, 2019; Krause et al., 2020). This dynamic nature of flexible MOFs makes them attractive for several potential applications such as sensors, molecular separation, gas storage, drug delivery, and catalysis (Mason et al., 2015; Majewski et al., 2017; Allendorf et al., 2020; Hou et al., 2020; Semrau et al., 2020; Wang et al., 2020). Applications of MOFs, however, often require material downsizing, especially for integration into the operating systems. The influence of the crystal downsizing on the structural transition characteristics in classical materials (alloys, organic compounds) stimulated by temperature or mechanical pressure was intensively studied in the past, showing pronounced particle size dependence (Li et al., 2016a; Anwar and Zahn, 2017). The postulated observations could be rationalized, considering the internal pressure and surface energy differences between polymorphs, confinement effects, and nucleation theory (Yang, 1999; Nièpce and Pizzagalli, 2007; Li et al., 2016a; Anwar and Zahn, 2017).

Recently, the impact of the crystal size on functional properties of MOFs has also received considerable attention (Sakata et al., 2013; Zhang et al., 2014; Li et al., 2016b; Saitoh et al., 2016; Tian et al., 2016). It was recognized that, by changing the particle size, the intermolecular interaction between the adsorbent and adsorbate, the catalytic performance of the MOF, and the magnetic properties can be tuned (Hatakeyama et al., 2011; Kiyonaga et al., 2015; Ehrling et al., 2021).

The findings regarding the particle size-dependent phenomena in flexible MOFs are summarized in a recent publication (Ehrling et al., 2021). However, only a few studies dedicated to the crystal shape and its role on the characteristics of structural transition have been published so far (Watanabe et al., 2017; Zhang et al., 2017). In flexible MOFs, where the transitions are often adsorption driven, the transition characteristics may be influenced not only by the particle size but also by the crystal habit. The pore structure and pore connectivity, as well as associated pore accessibility, may determine the adsorption-induced phase transitions in porous materials. An important aspect, in particular for gate opening systems, is that the activation barrier for gate opening is mechanistically connected to the entry channel of the adsorptive into the framework. In this sense, nucleation is expected to begin on the outer surface of the crystal, and the morphology is expected to affect nucleation barriers significantly for a given adsorptive.

In this study, we report the influence of crystal size and morphology on gate opening transition in switchable DUT-8(Ni), intending to identify the crystal faces significantly affecting the gate opening pressure. DUT-8(Ni) is a pillared layer MOF, consisting of Ni_2_-paddle wheels (PWs), bridged by 2,6-naphthalene dicarboxylate (2,6-ndc) linkers to form 2D layers. Ni_2_-PWs are interconnected by 1,4-diazabicyclo[2,2,2]-octane (dabco) ligands into a three-dimensional [Ni_2_(2,6-ndc)_2_(dabco)]_n_ framework. Upon the guest-molecule removal, the open pore structure (op), containing guest molecules transforms to the closed pore structure (cp) (Bon et al., 2015a). Adsorption of guest species, such as nitrogen at 77 K, induces the reverse transition and the opening of the crystal structure. The unit cell volume difference of cp and op phases is 254% (Bon et al., 2015b).

Crystal downsizing to the submicron range enables the trapping of the material in a metastable, open, solvent-free phase and transforming it to a microporous rigid adsorbent (Kavoosi et al., 2017). Previous studies have shown that the crystal size of DUT-8(Ni) can be tuned by micromixer synthesis, controlling the nucleation process and crystal growth by contact time of starting materials and aging time (Miura et al., 2017). The variation of the crystal size in the micrometer range leads to changes in the slope of nitrogen adsorption isotherms. In the present contribution, the crystal size and the shape were manipulated by synthetic parameters, such as the crystallization method and chemical-modulating agents, which facilitated to control not only the size of the crystals but also the crystal habit.

Two different types of modulators were applied: (i) small molecules possessing functional groups chemically related to the organic building blocks of the MOF and (ii) macromolecules, bearing the same functionality (Han et al., 2019). To correlate the gate opening characteristics of the isotherms with the size and morphology of the DUT-8(Ni) crystals, we analyzed the materials using scanning electron microscopy (SEM), electron diffraction, and nitrogen physisorption at 77 K.

## Materials and Methods

### Chemicals

All chemicals were used as received without further purification. Ni(NO_3_)_2_·6H_2_O (97%), 2,6-H_2_ndc (99%), dabco (99%), polyacrylic acid (PAA; M_w_ = 1,800), and dry methanol were purchased from Sigma Aldrich and *N,N*-dimethylformamide (DMF) of reagent grade purity from Fisher Chemical. Acetic acid (100%) and pyridine (99%), extra pure, were purchased from Carl Roth and Acros Organics, respectively.

### Synthesis

#### Synthesis Without Modulating Agents

##### Samples A, B, and C

In a typical synthesis, Ni(NO_3_)_2_·6H_2_O (0.407 g, 1.4 mmol) was dissolved in 6 ml DMF, 2,6-H_2_ndc (0.303 g, 1.4 mmol) was dissolved in 15 ml DMF, and dabco (0.1 g, 0.9 mmol) was dissolved in 9 ml methanol (MeOH) using ultrasonic bath and mixed (Kavoosi et al., 2017). Samples A, B, and C were synthesized using the same composition of the reaction mixture but utilizing different crystallization conditions. The reaction mixture for sample A was heated in a Teflon-lined autoclave under static conditions for 48 h at 393 K. Afterwards, the mother liquor was removed by centrifugation, and crystals were washed several times with DMF. For the preparation of sample B, the autoclave was rotated for 48 h using a BINDER oven at 393 K. The reaction mixture of sample C was heated by microwave irradiation of 150 W for 30 s.

#### Small Molecules Possessing Functional Groups Chemically Related to the Organic Building Blocks as Modulators (i)

**Figure 1:**
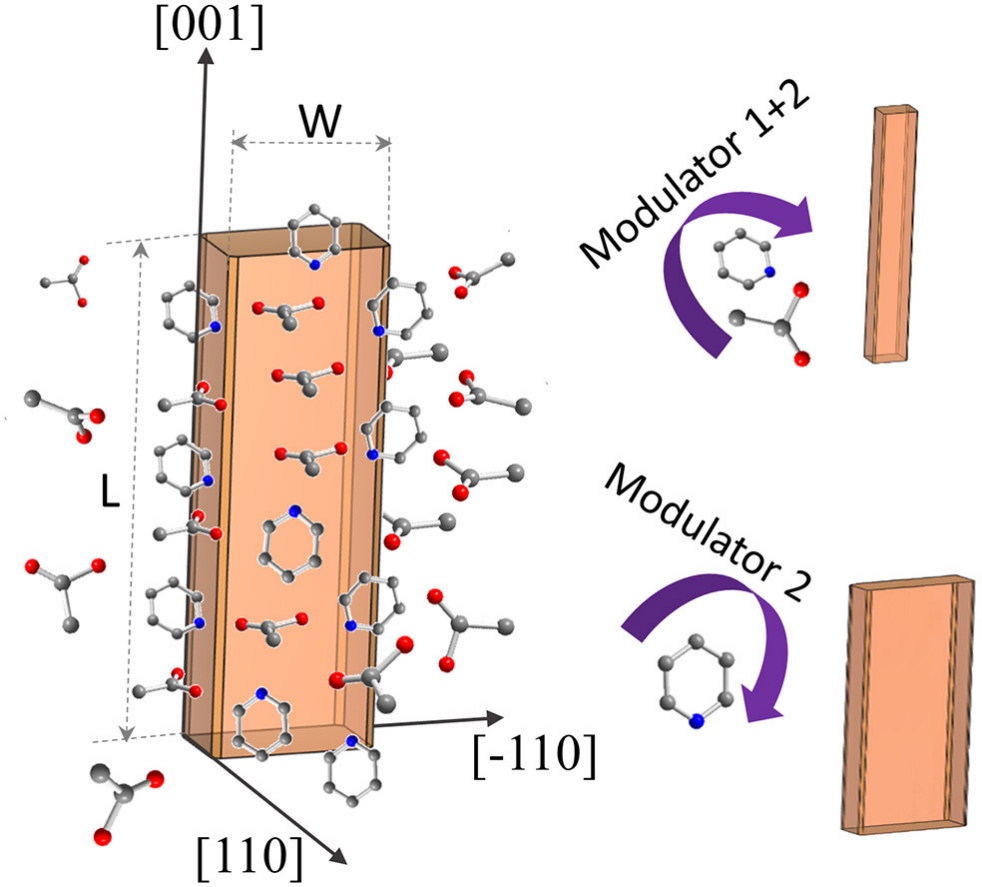
Schematic representation of DUT-8(Ni) coordination modulation by acetic acid and pyridine.

The synthesis procedure described for the synthesis of submicron-sized, rigid DUT-8(Ni) crystals (Kavoosi et al., 2017) was adopted for morphology modification using acetic acid (modulator 1) and pyridine (modulator 2) as capping agents (Figure 1).

##### Sample D

All chemicals were dissolved separately: Ni(NO_3_)_2_·6H_2_O (0.145 g, 0.5 mmol) in 1.5 ml DMF, 2,6-H_2_ndc (0.096 g, 0.44 mmol) in 7 ml DMF, and dabco (0.112 g, 1 mmol) in 1.5 ml DMF using an ultrasound bath for 10 min before mixing. To synthesize plates, solutions of 2,6-H_2_ndc and dabco were mixed with the subsequent addition of 0.05 ml pyridine (modulator 2). Afterward, nickel nitrate solution was added to the reaction mixture. A cloudy suspension was obtained after mixing all the chemicals, which was transferred to a Teflon vessel (50 ml) and heated at 393 K in autoclave for 24 h. After synthesis, the mother liquor was removed by centrifugation, and the resulting particles were washed several times with DMF.

##### Sample E

All the chemicals were dissolved separately: Ni(NO_3_)_2_·6H_2_O (0.145 g, 0.5 mmol) in 1.5 ml DMF, 2,6-H_2_ndc (0.096 g, 0.44 mmol) in 7 ml DMF, and dabco (0.112 g, 1 mmol) in 1.5 ml DMF, using an ultrasound bath before mixing for 10 min. The solutions of 2,6-H_2_ndc and dabco were combined with the subsequent addition of 0.05 ml pyridine (modulator 2). Afterward, a nickel nitrate solution, containing 0.05 ml of acetic acid (modulator 1), was added to the reaction mixture. A cloudy suspension was obtained after mixing all the chemicals, which was transferred to a Teflon vessel (50 ml) and heated at 393 K in autoclave for 24 h. After synthesis, the mother liquor was removed by centrifugation, and the resulting particles were washed several times with fresh DMF.

#### Polyacrylic Acid as Modulator (ii)

##### Samples PAA_1, PAA_2, and PAA_3

Synthesis of flexible DUT-8(Ni), reported by Kavoosi et al. (2017), was adopted for morphology modification of crystals, using PAA (modulator 3). Typically, Ni(NO_3_)_2_·6H_2_O (0.204 g, 0.7 mmol) was dissolved in 3 ml DMF, 2,6-H_2_ndc (0.152 g, 0.7 mmol) was dissolved in 7.5 ml DMF, and dabco (0.05 mg, 0.45 mmol) was dissolved in 4.5 ml methanol. The three resulting solutions were combined prior to the addition of PAA (0.001 g). The mixture was sonicated for 10 min, transferred into a Teflon vessel (50 ml in volume), and heated in an autoclave at 393 K for 48 h. The resulting crystals were washed with DMF several times. The sample is termed PAA_1. In the following syntheses, the amount of PAA was varied: sample PAA_2 was synthesized under the addition of 0.005 g, and sample PAA_3 was synthesized under the addition of 0.01 g of modulator 3.

Thermogravimetric analysis, IR-, and NMR-spectroscopy measurements were performed (Supplementary Figures 5–7), confirming the absence of residual modulators in D, E, PAA_1, PAA_2, and PAA_3, at least within the limit of quantitation of the methods.

### Solvent Removal

*N,N*-dimethylformamide used in the synthesis was exchanged first by dichloromethane (DCM) for 3 days. Afterward, the crystals were filtered under argon flow, and the solvent was removed under vacuum at 423 K for 16 h.

### Characterization Methods

#### Powder X-Ray Diffraction

Powder X-ray diffraction patterns were obtained at room temperature on an STOE STADI P diffractometer, using Cu–Kα1 radiation (λ = 1.5406 Å) and a 2D detector (Mythen, Dectris). All measurements were performed in transmission geometry using a rotating flatbed sample holder, 2Θ resolution of 3.12°, and an exposition time of 120 s per step.

#### Physisorption

Volumetric nitrogen physisorption measurements were performed on BELSORP-MAX, Autosorb IQ, or Quadrasorb apparatuses, using liquid nitrogen to reach the temperatures of measurements of 77 K. Prior to the adsorption measurement, the samples were evacuated at 298 K for 16 h.

#### Scanning Electron Microscopy

Scanning electron microscopy measurements were carried out by taking a secondary electron using 2–5 kV acceleration voltage and a working distance of 4–14 mm on a SU8020 from Hitachi. Before the measurement, the samples were sputtered with Au to enhance surface conductivity. SEM images of the crystals obtained were analyzed by ImageJ software and the corresponding crystal size distributions were calculated (ImajeJ, 2021). Polydispersity index (PDI) was calculated as follows: PDI = SD^2^/mean^2^ [SD, standard deviation; mean, the averaged value of the crystal size (μm)].

#### Thermogravimetric Analysis

Thermogravimetric analysis was performed in synthetic air flow in a temperature range from 298 to 1,273 K with a heating rate of 5 K min^−1^, using an STA 409 PC from NETZSCH Company.

#### IR Spectroscopy

Fourier transform (FT-IR) ATR spectra were collected at 2-cm^−1^ resolution, ranging from 4,000 to 400 cm^−1^, using a Bruker Vertex 70 Alpha instrument equipped with an ATR accessory (diamond crystal) and placed inside the device.

#### NMR Spectroscopy

^1^H NMR experiments in solution were performed, using a Bruker AVANCE 300 MHz (300 MHz, 282 MHz) spectrometer. The samples were dissolved in DCl/D_2_O in DMSO-d6.

#### 3D Electron Diffraction (3D ED)

3D ED data were collected using a Termofisher TITAN TEM, equipped with a 2 k Gatan MSC camera, in nanodiffraction geometry with the C2 condenser aperture of 50 μm, and the effective beam diameter on the sample of 1 μm. Acceleration voltage was 300 kV.

## Results and Discussion

### Modulation of Crystal Size

To systematically vary the size of the DUT-8 crystals, the synthetic procedure was altered in crystallization conditions, without the application of additional chemicals or changing the reaction mixture composition. Thus, samples A, B, and C were synthesized under static conditions, or by shaking the reaction vessel, or under rapid heating by microwave irradiation, respectively.

**Figure 2:**
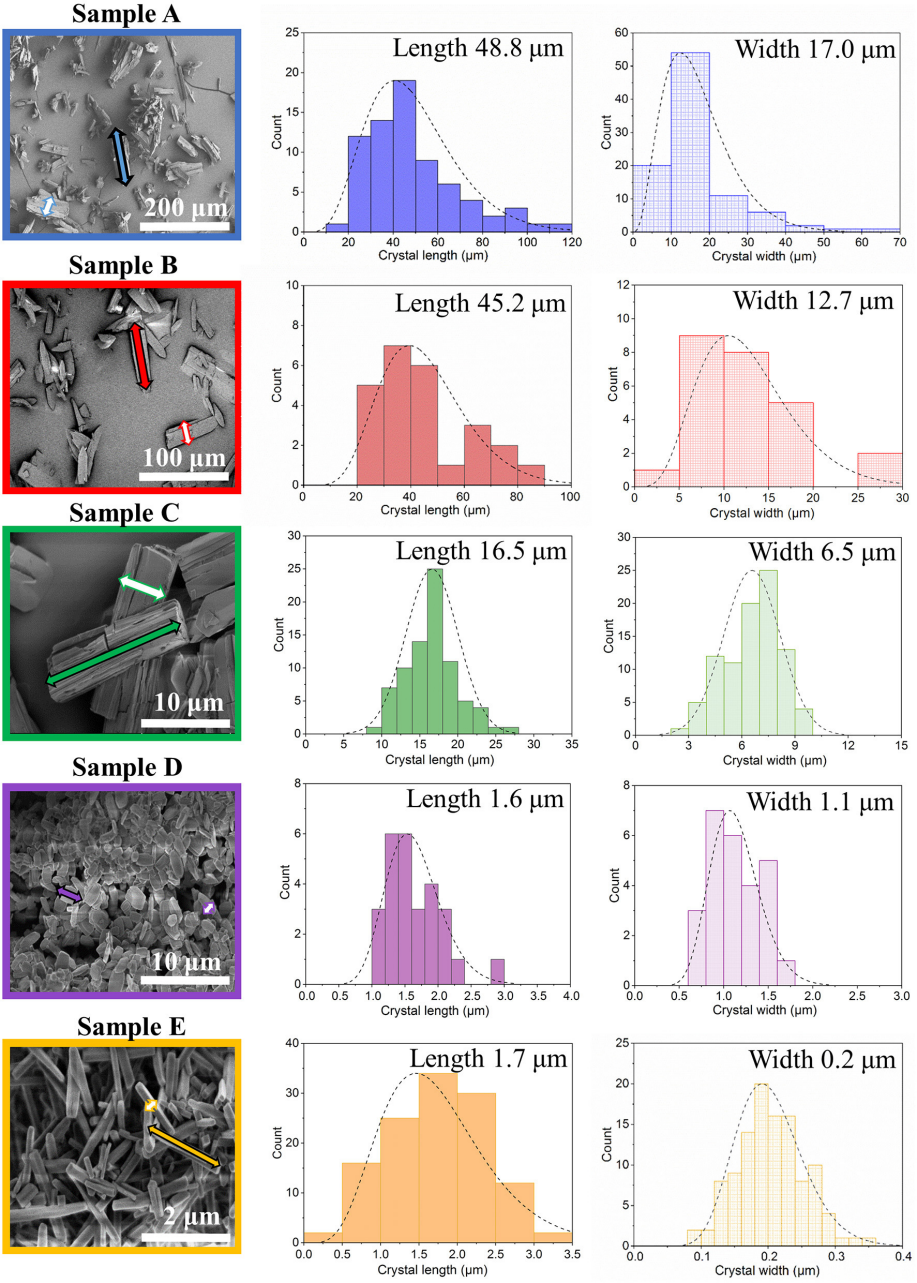
SEM images and particle size distributions (length marked as a filled arrow in SEM and width marked as an empty arrow in SEM) of the investigated samples.

Scanning electron microscopy images of the crystals obtained were analyzed by ImageJ software, and the corresponding crystal size distributions were calculated (Figure 2, Supplementary Figure 9, ESI). Due to the rod-like morphology of DUT-8(Ni), we decided to use the length (L)-to-width (W) ratio (aspect ratio) as an appropriate parameter to monitor the changes in the habit, in addition to the length (as it was done previously to monitor the crystal size). In this study, we also have paid increased attention to the crystal width. The DUT-8(Ni) compound, containing DMF in the pores, crystallizes in the monoclinic space group with the cell parameters *a* = 18.576(3), *b* = 18.408(2), *c* = 9.3574(13), and β = 97.545(9) (Petkov et al., 2019). Thus, the length of the rod-like crystal corresponds to the size along the crystallographic *c* direction running along the pillars. The crystal width, on his part, is defined by the size along *a*–*b* crystallographic directions (plain of the layers).

The SEM image of sample A, which was heated for 48 h (conventional DUT-8(Ni) synthesis), highlights that the length of crystals varies between 20 and 120 μm, with a mean of 48 μm. The mean width of crystals is 17 μm. The SEM images of sample B confirm the influence of the reaction mixture, stirring on the crystal size, decreasing the length of the crystals to 45 μm, and width to 12 μm. Microwave-assisted synthesis leads to further crystal downsizing, yielding crystals with 16 μm in length and 6 μm in width in average. Thus, different synthesis conditions lead to a successive decrease of the length and the width of the crystal simultaneously, leading to the insignificant differences in the aspect ratio (ca. 3 on average) of these crystals (Table 1).

**Table 1:** Characteristics of the investigated samples.

**Sample**	**APHM**	**Length (L)/μm**	**RSD/%**	**PDI**	**Width (W)/μm**	**RSD/%**	**PDI**	**Aspect ratio (L/W)**	**Total pore volume/cm^3^ g^−1^**	**α_max_**
A	0.17	48.8	43.6	0.19	17.0	63.2	0.40	2.9	1.05	0.99
B	0.21	45.2	37.5	0.14	12.7	45.8	0.21	3.5	1.06	1
C	0.30	16.5	20.7	0.04	6.5	24.3	0.05	2.5	1.05	0.98
D	0.44	1.6	26.5	0.07	1.1	24.7	0.06	1.4	0.95	0.89
E	0.69	1.7	37.1	0.13	0.2	24.1	0.06	8.6	0.90	0.85
PAA_1	0.19	52.9	56.6	0.32	17.1	50.8	0.25	3.1	1.03	0.97
PAA_2	0.23	55.0	18.3	0.03	15.2	23.0	0.05	3.3	1.06	1
PAA_3	0.26	53.3	16.7	0.03	8.0	26.7	0.07	6.7	1.03	0.97

*RSD, relative standard deviation; PDI, polydispersity index; APHM estimated for the normalized isotherms (Supplementary Figure 3A)*.

The addition of monodentate ligands that mimic the functionality of the corresponding linker in the synthesis solution (coordination modulation) is widely used to control the particle size of MOFs (Forgan, 2020). For example, Kitagawa and co-workers have utilized the “coordination modulation” approach to control the crystal size and shape in a non-switchable pillared-layer MOF [Cu_2_(1,4-ndc)_2_(dabco)]_n_(1,4-ndc−1,4-naphthalenedicarboxylate) (Tsuruoka et al., 2009). This material can be regarded as isoreticular to switchable DUT-8(Ni), presenting crystal faces terminated by either carboxylate units from the carboxylic linkers or nitrogen atoms from the dabco pillar (Figure 1). The different functionalities of the linker and pillar allow for selective capping of specific faces by the addition of modulators, complementary to the chemistry at particular crystal faces. The addition of molecules containing carboxylic groups (Modulator 1) to the synthesis mixture may result in the formation of needles if the crystal growth rate on capped faces is decreased. In the case of DUT-8(Ni), the addition of acetic acid only resulted in the crystals of increased size, and no needles were obtained. Modulation with pyridine (modulator 2), where the N-donor is expected to coordinate to the {001} crystal faces (Figure 1), reported to produce nanosheets, could be adapted to the DUT-8(Ni) system (sample D). Synthesis with pyridine as modulator produced uniform plate-shaped crystals. However, in contrast to the expectations, the smallest dimension of the crystal corresponds not to the [001], but to the [110] (or [−110]) direction (Figure 1). The length (size in crystallographic *c* direction) of crystals in D is significantly decreased to 1.6 μm (in comparison with A, B, and C); meanwhile, the expansion in the *a*–*b*-plane is 0.3 × 1.1 μm, lowering the aspect ratio to 1.4 and resulting in platelets.

Modulation with both pyridine and acetic acid, reported to result in a particle size decrease and formation of nanocubes (Pham et al., 2012) for [Cu_2_(1,4-ndc)_2_(dabco)]_n_, yielded needles (sample E) in case of DUT-8(Ni). The crystal dimensions of needle-like sample E are 1.7 μm in length and 0.2 μm in width, rising the aspect ratio to 8.6 μm. The assignment of the crystallographic directions to the length and the width of crystal in samples D and E was performed by electron diffraction, using TEM (Sections 12 and 13 in ESI).

**Figure 3:**
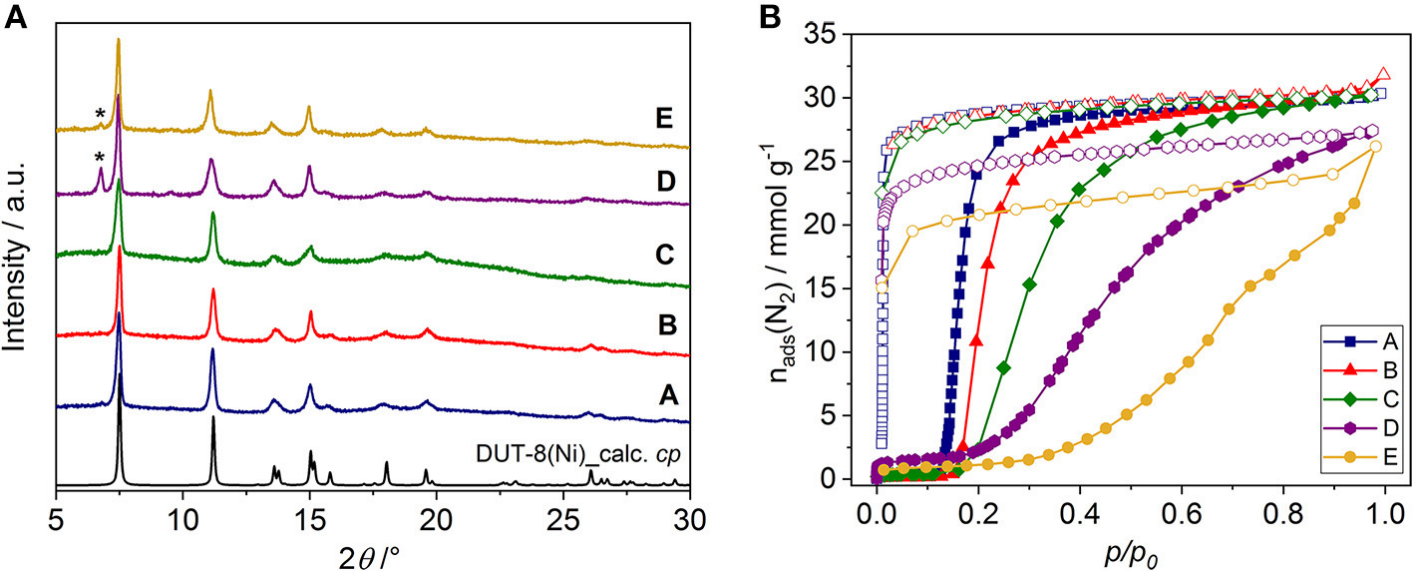
**(A)** PXRD patterns of DUT-8(Ni) samples in the desolvated state. [The theoretical pattern calculated from the single crystal structure (CCDC-1912233) is shown in black.] Peaks corresponding to the op phase are marked (*). **(B)** Nitrogen physisorption isotherms at 77 K for the samples investigated (for semilogarithmic plots, see Supplementary Figure 3B, ESI). Closed symbols: adsorption; open symbols: desorption.

In all cases, phase pure DUT-8(Ni) compounds were obtained according to PXRD analysis (Supplementary Figure 1, ESI). After the synthesis, all samples were treated uniformly, i.e., washed with dichloromethane and desolvated in a vacuum at 423 K. The solvent removal leads to framework contraction according to PXRD patterns (Figure 3A), which are in good agreement with the theoretical pattern of DUT-8(Ni) in the cp phase. The PXRD patterns of samples D and E, however, contain small peaks of the remaining op phase, pointing on the part of the sample falls below the critical size to show flexibility (Kavoosi et al., 2017; Miura et al., 2017).

### Analysis of Adsorption Behavior With Respect to Crystal Size and Habit

The switching behavior of all samples was investigated by nitrogen adsorption at 77 K, showing that the crystals habit significantly influences the switching and thereby adsorption behavior of DUT-8(Ni) (Figure 3B, Supplementary Figure 3).

To understand the effects on the isotherm and the relationship between the size, morphology, and isotherm shape, it is important to identify characteristic quantities, which can be extracted from the isotherm and related to the sample characteristics, thermodynamics, and kinetics of the switching process. Therefore, as the main characteristics for the discussion and comparison of the gate pressure isotherms, typical for DUT-8(Ni), we will use the following quantities:

(i) Uptake at highest relative pressure reached in the adsorption experiment (or in plateau). It represents the specific pore volume of the particular sample. In relation to the theoretical pore volume, it reflects the fraction of crystallites (α_max_) transformed to the op phase at given conditions. In the powdered samples, the phase transformation often does not go to completion (Anwar and Zahn, 2017). The reason here is that some of the crystallites cannot be stimulated to open at a particular pressure because of the high activation barrier for opening. Higher pressures (Krylov et al., 2020) or adsorption enthalpies (Bon et al., 2015a) would enable the stimulation of these crystals, exhibiting higher activation energy, forcing additional crystallites to transform, but the maximum pressure in the adsorption experiments is limited to the saturation pressure of the chosen adsorptive (1 atm at 77 K).

The samples A, B, and C reach almost full nitrogen uptake at 77 K after opening, indicating complete phase transition in almost all crystallites (Table 1). The isotherms of samples D and E, however, do not reach a plateau upon adsorption up to high relative pressures, indicating that a part of the sample remains in the cp phase (Miura et al., 2017). Consequently, the specific pore volume and α_max_ for these samples are lower than expected for a complete transition.

(ii) Relative adsorption pressure at half maximum uptake (APHM) as a measure of the average gate opening activation energy (ΔG^*^).

In general, the first-order phase transitions show a hysteresis, because of the kinetic barriers controlling the nucleation of the new phase (Evans et al., 2020). The hysteresis width depends on the barrier and the rate at which the thermodynamic conditions change within a given experiment (Anwar and Zahn, 2017). The ideal hysteresis loop of single crystallite (single grain) in the adsorption isotherm would have a rectangular shape corresponding to the Preisach model (Mayergoyz, 2003), where the only change in porosity is due to structure transition events. The hysteresis loop will have a more complex shape for a powdered sample as an ensemble (arrangement), consisting of quasi “non-interacting grains.” Thus, the distribution of nucleation barriers of the individual grains controls the transition pressure spread over a wider pressure range. This is typically reflected by the less-steep adsorption branch in the gating region (Ehrling et al., 2021). The first derivative of the adsorption branch of the nitrogen physisorption isotherm reflects the distribution of transition pressures over the sample and, correspondingly, the distribution of gate opening activation energies (**Figure 5**, Supplementary Figures 11 and 12, ESI).

So, the APHM represents the average width or breadth of the hysteresis (taking into account non-significant changes in the gate closing pressure) and, thereby, the average activation energy of the grains within the sample.

**Figure 4:**
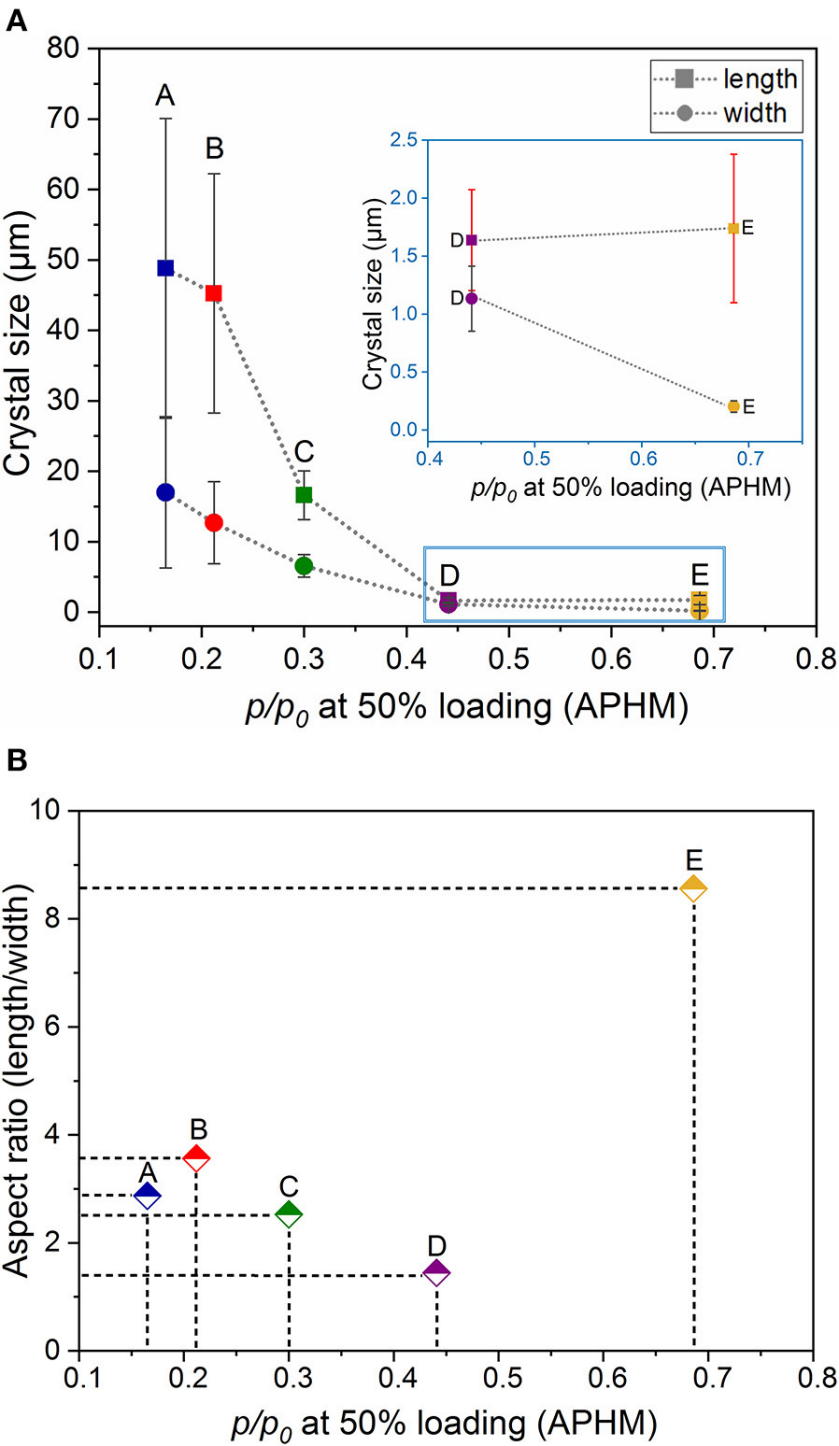
Relationship between the relative adsorption pressure at half maximum uptake (APHM) and the crystal dimensions (length and width) **(A)** and shape (aspect ratio) **(B)**.

Dependence of the relative adsorption pressure at half maximum uptake from the crystal dimensions (length and width) and shape (aspect ratio) is summarized in Table 1 and is shown in Figures 4A,B. It can be clearly seen that the aspect ratio is not the determining parameter, influencing the gate opening pressure. The APHM changes also do not systematically follow the changes in the crystal length. The APHM for sample E is larger than that for sample D, although the crystals are almost equal in length (Supplementary Figure 18, ESI). Analyzing the trend for all the samples, and especially the APHMs for the samples D and E (Figure 4A inset), it is evident that the systematic changes in the width of the crystals lead to the systematic APHM changes. Thus, obviously, the crystal width determines the gate opening pressure in DUT-8(Ni).

To confirm the dominant influence of particle width on dynamic properties upon adsorption of nitrogen at 77 K ones more, an additional crystal series was prepared, utilizing PAA as a modulator (Supplementary Figures 1, 2, ESI). The same chemicals present in the synthetic mixture ensure the same terminating groups on the crystal outer surface, which is not necessarily the case if modulators, differing in functional groups, are used to influence the morphology (as it is the case for samples A, B, and C vs. D vs. E). The impact of the surface termination on the flexibility of DUT-8(Ni), however, has been recently analyzed by the group of Düren (Thompson et al., 2020).

In PAA-modulated syntheses (PAA_1, PAA_2, and PAA_3), a low amount of a polymer does not modify the morphology, yielding needles (PAA_1) or intergrowth of needles with a wheat sheaf habit (in samples PAA_2 and PAA_3) (Supplementary Figure 4, ESI). With regard to crystal dimensions, the length was maintained approximately in the same range (53–55 μm, Supplementary Figure 4, ESI), while the critical parameter (width) varies from 17 to 8 μm. The change in crystal width affects the adsorption profile, which is reflected in the APHM shift to the higher values, with decreasing width of the needles (Supplementary Figure 3C, ESI), confirming the trend observed in the first A–E series.

**Figure 5:**
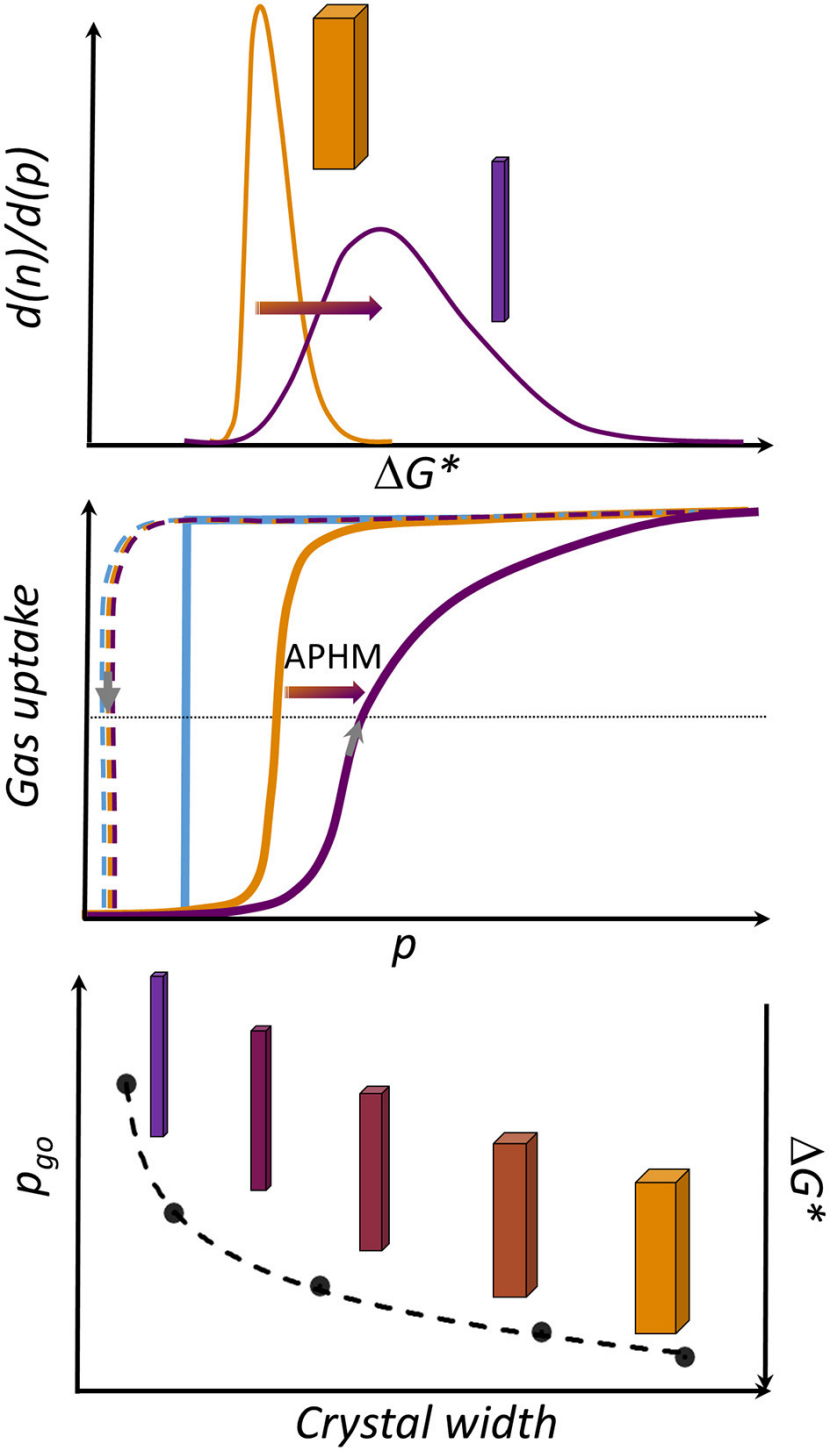
Overview of crystal-size-dependent behavior in DUT-8(Ni). **(Top)** Distribution of activation energies (ΔG*) in the sample, depending on the crystal width (W). **(Middle)** Rectangular gate opening isotherm (blue) corresponding to an ideal single grain-switching event. Orange: schematic representation of the isotherm usually obtained for macro-sized crystals. Violet: typical isotherm characteristic for micron-sized particles (adsorption: solid line, desorption: dashed line). **(Bottom)** Dependence of the gate opening pressure from W.

The changes in the APHM with width, considering all samples from this study, as well as a reference sample from Miura et al. (2017), are not linear and follow logarithmic function in the range of the crystal sizes investigated (Supplementary Figure 8, ESI). Moreover, the steepness of the adsorption branch in the gating region decreases with decreasing crystal size, pointing at the significant broader activation energies distribution in the small grains in comparison to the large crystallites (Figure 5).

Taking into account the decreasing α_max_ for the small crystallites, the crystal width approaches a certain critical value where the transition is completely suppressed due to the height of the barrier (ΔG^*^), which cannot be overcome at given conditions. Another potential explanation for the observations is based on thermodynamic arguments, most importantly the differences in free energies of the empty cp and op frameworks (Δ*F* = *F*_*cp*_*-F*_*op*_), originating from differences in surface energy and, probably, less important gradients of the adsorbed phase (Ehrling et al., 2021).

**Figure 6:**
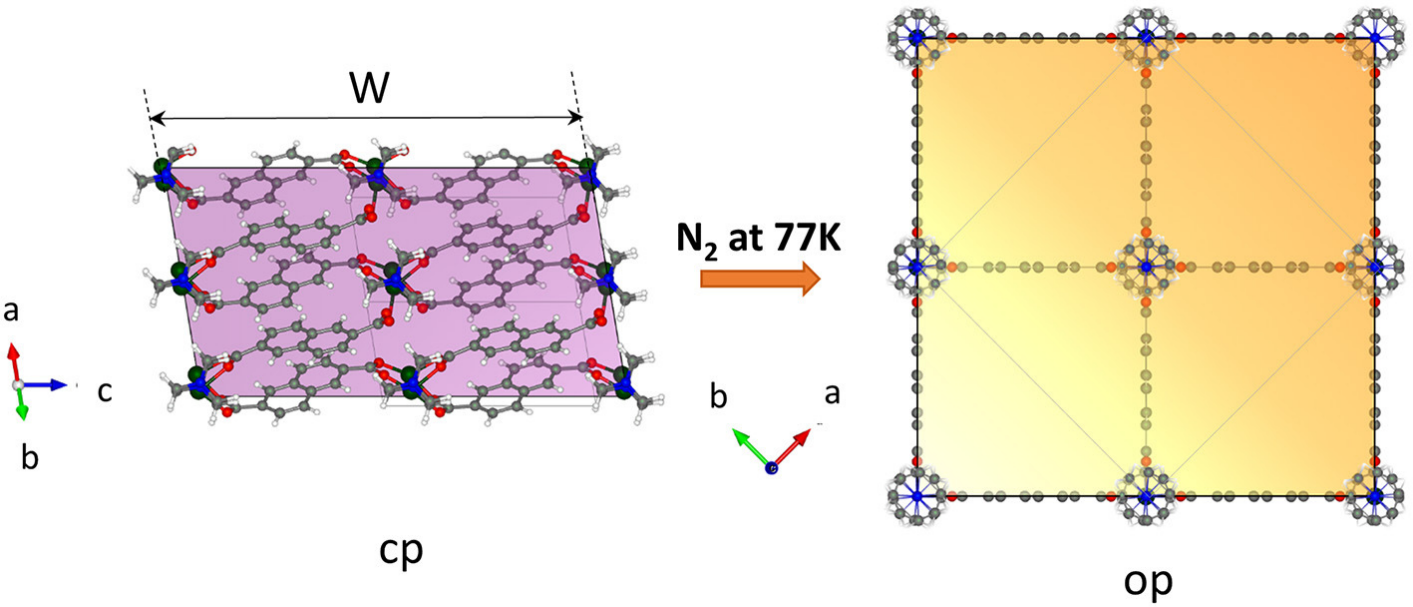
View on the crystal in the cp and op phase along the pore channel (generated by the VESTA 3.5.5 software) (Momma and Izumi, 2011). The colored face is the face representing the entry of the channel-like pore structure.

Important arguments of why the crystal width represents the critical value for gate opening can be discussed by analyzing the relationship between the crystal morphology and the structure of the pores. DUT-8 in the op phase has square channels running along the crystallographic *c* direction (Supplementary Figure 10, ESI). The walls of the channels are built mainly from aromatic naphthalene cores of 2,6-ndc linkers. The corners are formed by rows of alternating paddle wheel–dabco units (Figure 6). The channels can be considered as virtually isolated since the analysis of the geometrical pore parameters of at least the static crystal structure suggests only small interconnection windows between the main channels (Supplementary Figure 10, ESI) accessible for guest molecules with 3.6 Å in diameter [kinetic diameter of nitrogen molecule (Mehio et al., 2014)].

Essentially, with decreasing width of the crystals, i.e., with reduced dimensions in the *a*–*b* plane (referring to the op structure), the surface on the top and the bottom of the crystal (area of {001} faces) where the molecules enter the channels is reduced. The reduced number of entry channels accessible for the molecules represents a “bottleneck” and reduces the initial energetic gain of the adsorption enthalpy, leading to an increased barrier for the guest-induced transformation.

The data obtained in this study show that the barrier for the transition from cp to op phase in DUT-8(Ni) increases with a decrease in the area of crystal faces essential for initiation of adsorption (faces perpendicular to the direction of channels), illustrating the importance of local nucleation starting at the crystal surface as the factor determining the kinetics of the switching process.

## Conclusion

The modulation approach was successfully used to affect the size and shape of the flexible pillared layer DUT-8(Ni) MOF crystals, showing gate opening behavior upon adsorption of nitrogen at 77 K, to investigate the dependency of structural transition from crystals' size and shape.

It could be explicitly seen that the crystal dimensions perpendicular to the axis of the channel-like pores influence the gate opening pressure. It indicates a higher activation barrier to result from reduced areas of facets, exposing the pore entry to the surface, providing a rationale for the observations, as the pores in DUT-8(Ni) are preferably accessible from the facets perpendicular to the channels. Since the width of the crystal determines the area of these faces, the crystal width (and not the lengths or the aspect ratio) controls the gate-opening process. The decrease in the crystal width also results in the less-steep adsorption branch in the gating region, pointing to the broadening of activation energy distribution.

## Data Availability Statement

The raw data supporting the conclusions of this article will be made available by the authors, without undue reservation.

## Author Contributions

IS and SK designed the experiments. LA, RE, and NK performed the synthetic work and adsorption experiments. SE collected and analyzed the SEM images. IS analyzed and interpreted all the data. TG and UK collected and analyzed the electron diffraction and TEM data. The first draft of the manuscript was written by IS and LA and all authors commented on previous versions of the manuscript. All authors read and approved the final manuscript.

## Conflict of Interest

The authors declare that the research was conducted in the absence of any commercial or financial relationships that could be construed as a potential conflict of interest.
