# Supplementary material for: Impact of Crystal Size and Morphology on Switchability Characteristics in Pillared-Layer Metal-Organic Framework DUT-8(Ni)
Source: Front Chem. 2021 May 13;9:674566. doi: 10.3389/fchem.2021.674566 (PMC8155289; doi:10.3389/fchem.2021.674566)
Supplement: Supplementary file 2 [file Data_Sheet_2.docx]

Supplementary Material

**Contents**

[1 PXRD patterns of as made samples in *N,N*-dimethylformamide 2](#_Toc70679723)

[2 PXRD patterns of desolvated DUT-8(Ni) samples 3](#_Toc70679724)

[3 Nitrogen physisorption 4](#_Toc70679725)

[4 Particle size analysis 6](#_Toc70679726)

[5 Thermogravimetric analysis (TGA) 7](#_Toc70679727)

[6 Infrared spectroscopy (IR) 8](#_Toc70679728)

[7 Nuclear magnetic resonance spectroscopy (NMR) 9](#_Toc70679729)

[8 Dependence of APHM on the crystal dimensions 12](#_Toc70679730)

[9 Scanning electron microscopy 13](#_Toc70679731)

[10 Analysis of the geometrical pore parameters of the static DUT-8(Ni) crystal structure 14](#_Toc70679732)

[11 Derivatives of adsorption isotherms 15](#_Toc70679733)

[12 Transmission electron microscopy (TEM) 17](#_Toc70679734)

[13 Electron diffraction 17](#_Toc70679735)

[14 Schematic representation of crystals dimensions 21](#_Toc70679736)

# PXRD patterns of as made samples in *N,N*-dimethylformamide


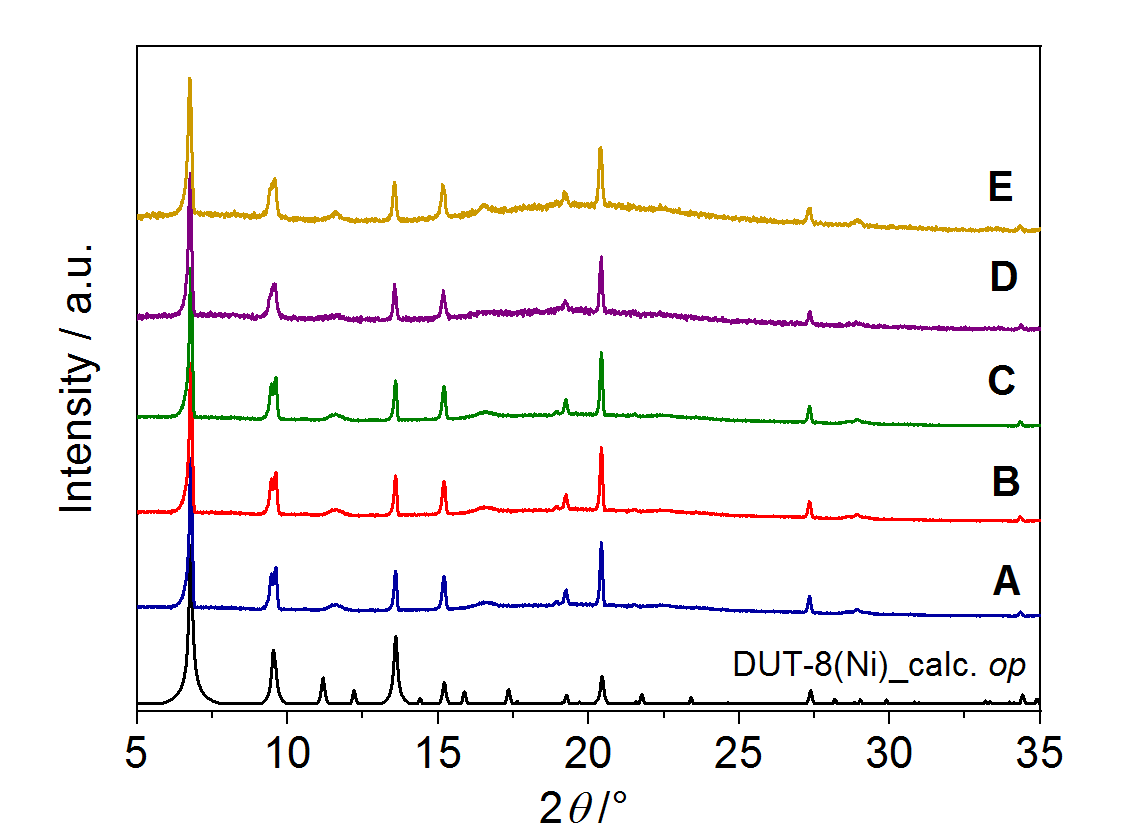


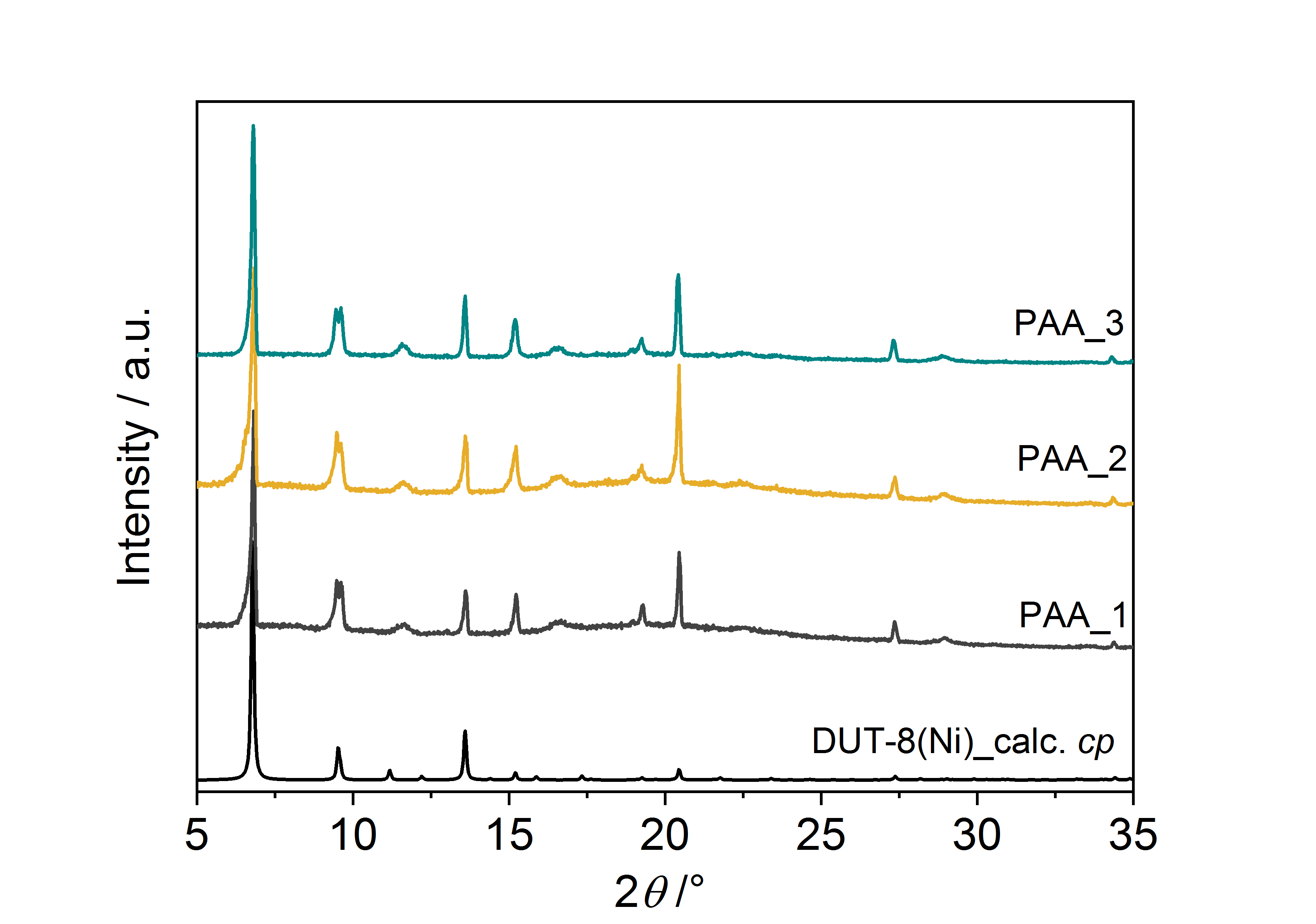


**Supplementary Figure 1.** PXRD patterns of the investigated as made DUT-8(Ni) samples.

# PXRD patterns of desolvated DUT-8(Ni) samples


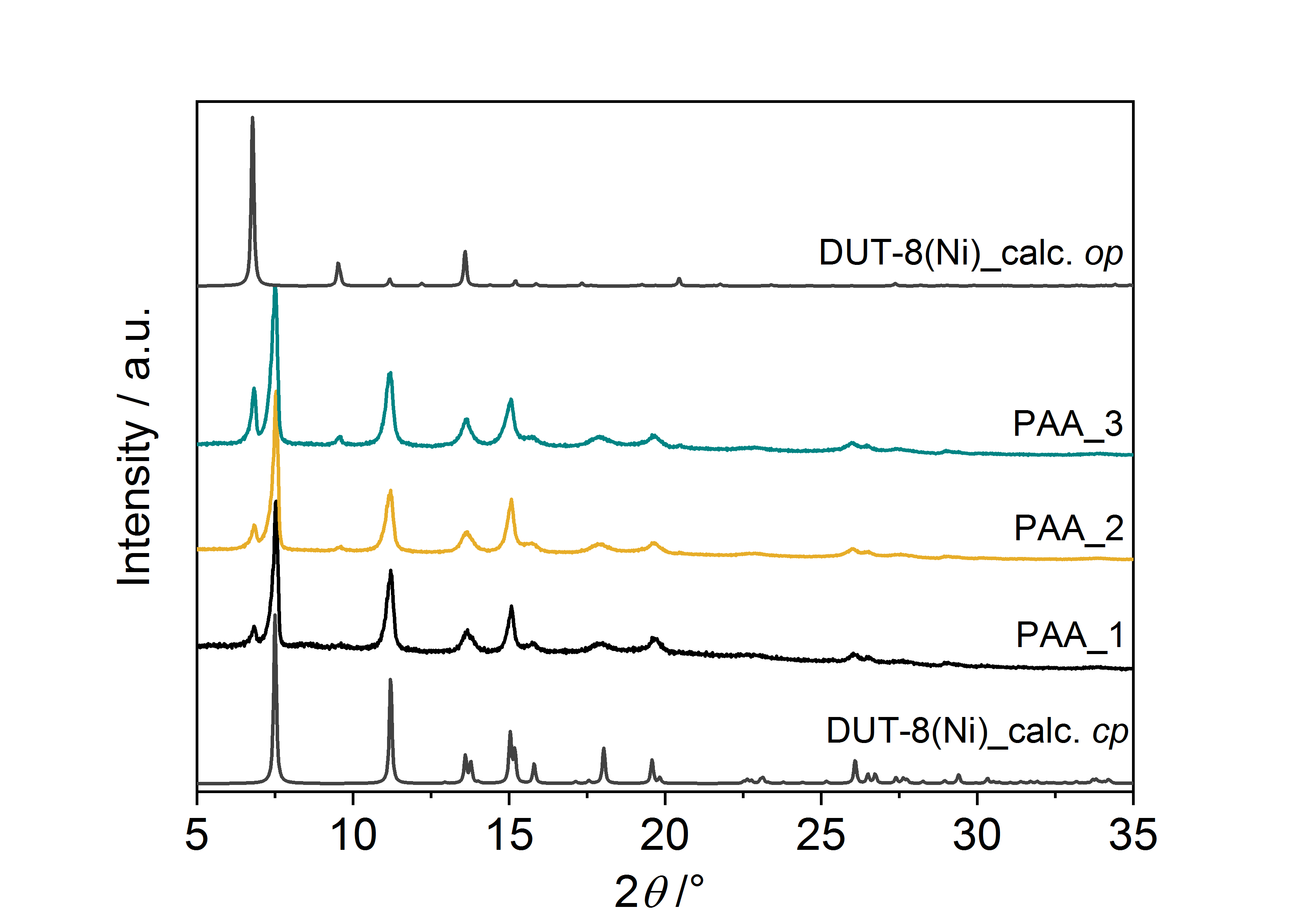


**Supplementary** **Figure 2.** PXRD patterns of DUT-8(Ni) modulated by polyacrylic acid (PAA) after desolvation.

# Nitrogen physisorption

Adsorption data are attached as ESI, in the form of Standard Adsorption Information files (AIF) (Evans et al., 2021).

(B)


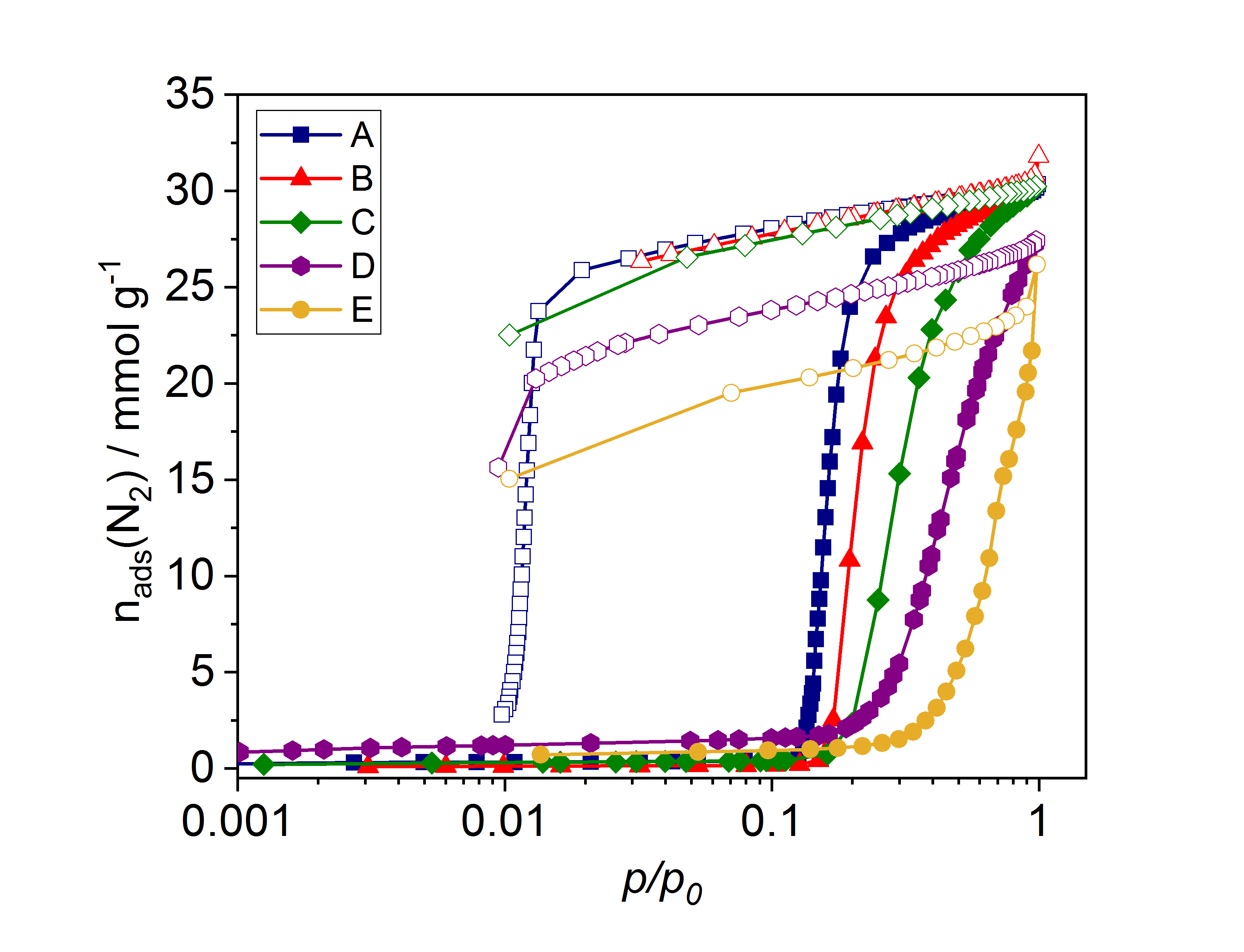

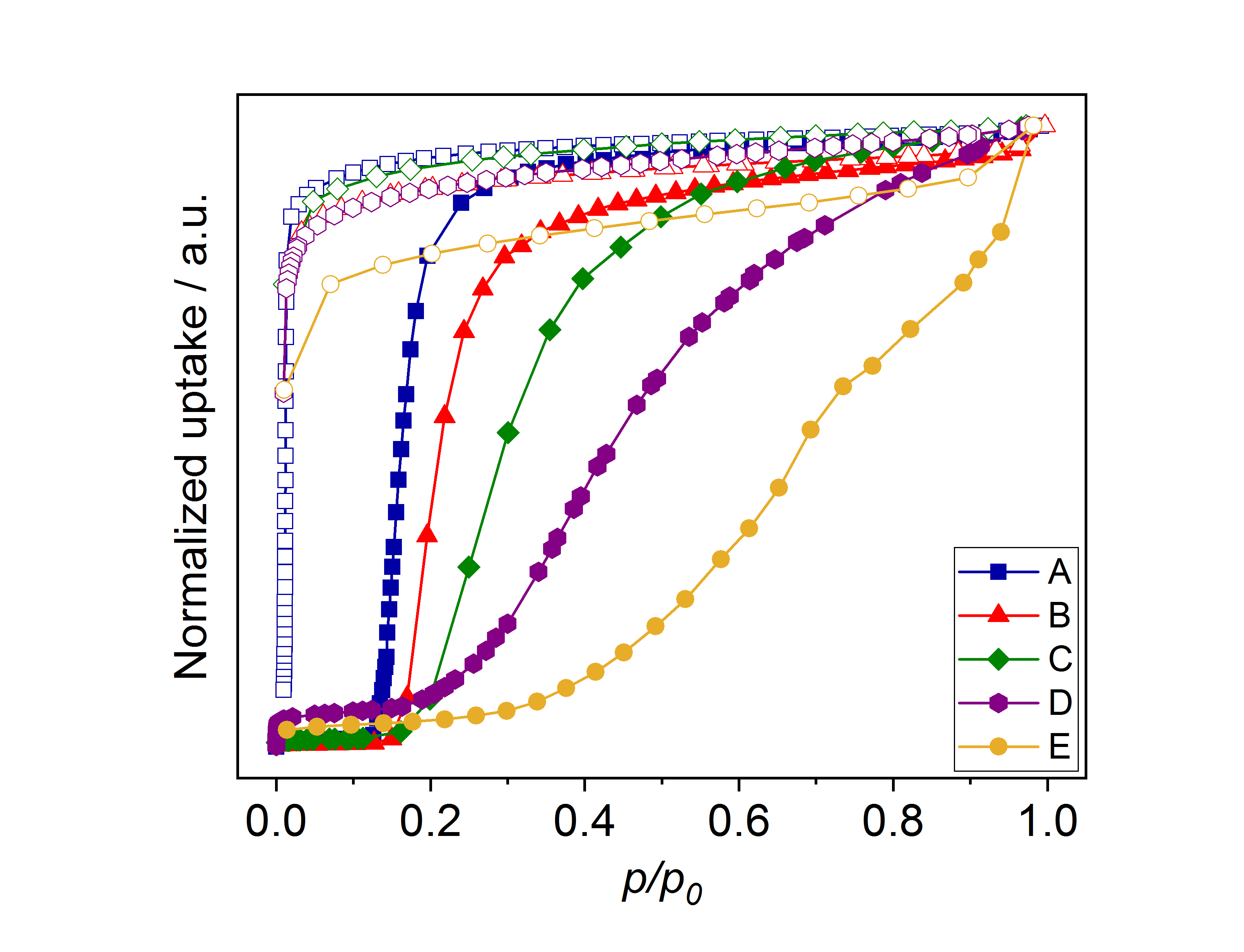


(A)

(C)


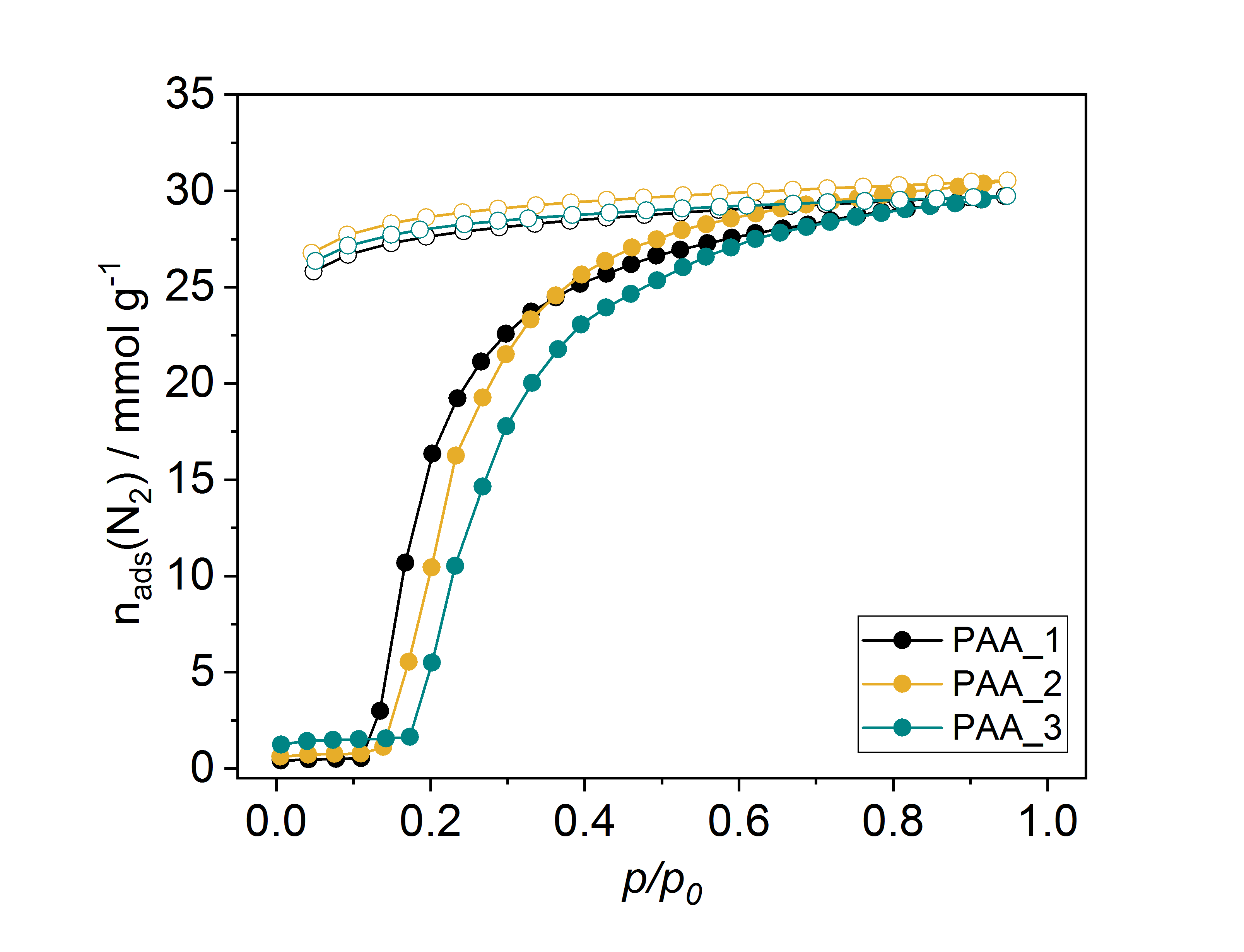


**Supplementary** **Figure 3**. Nitrogen physisorption isotherms at 77 K: (A) Uptake is normalized to the maximum value; (B) Semilogarithmic plot; (C) For modulated by polyacrylic acid (PAA). Closed symbols – adsorption, open symbols – desorption.

# Particle size analysis


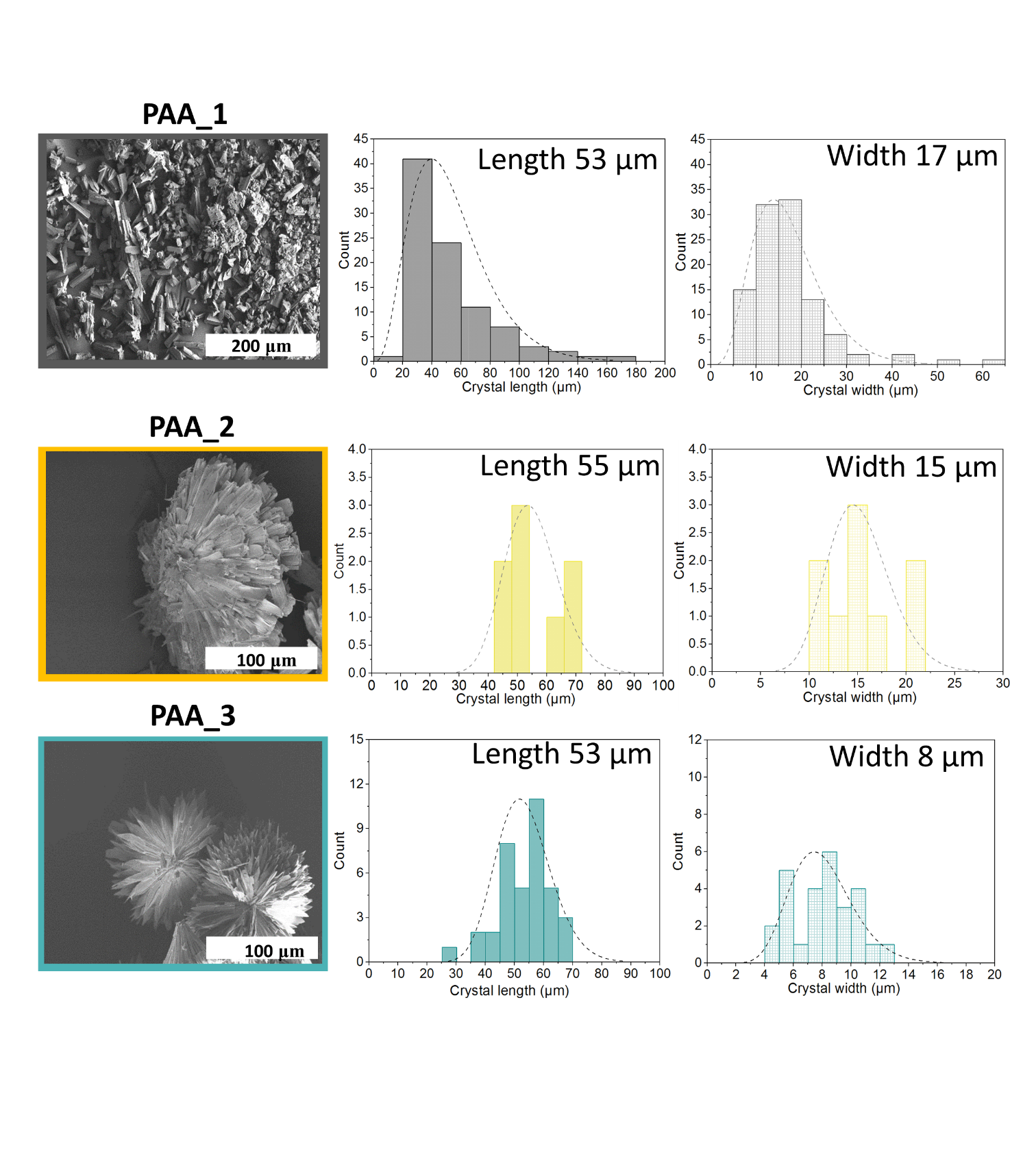


**Supplementary** **Figure 4.** SEM images and particle size (length, width) distributions for samples modulated by polyacrylic acid.

# Thermogravimetric analysis (TGA)


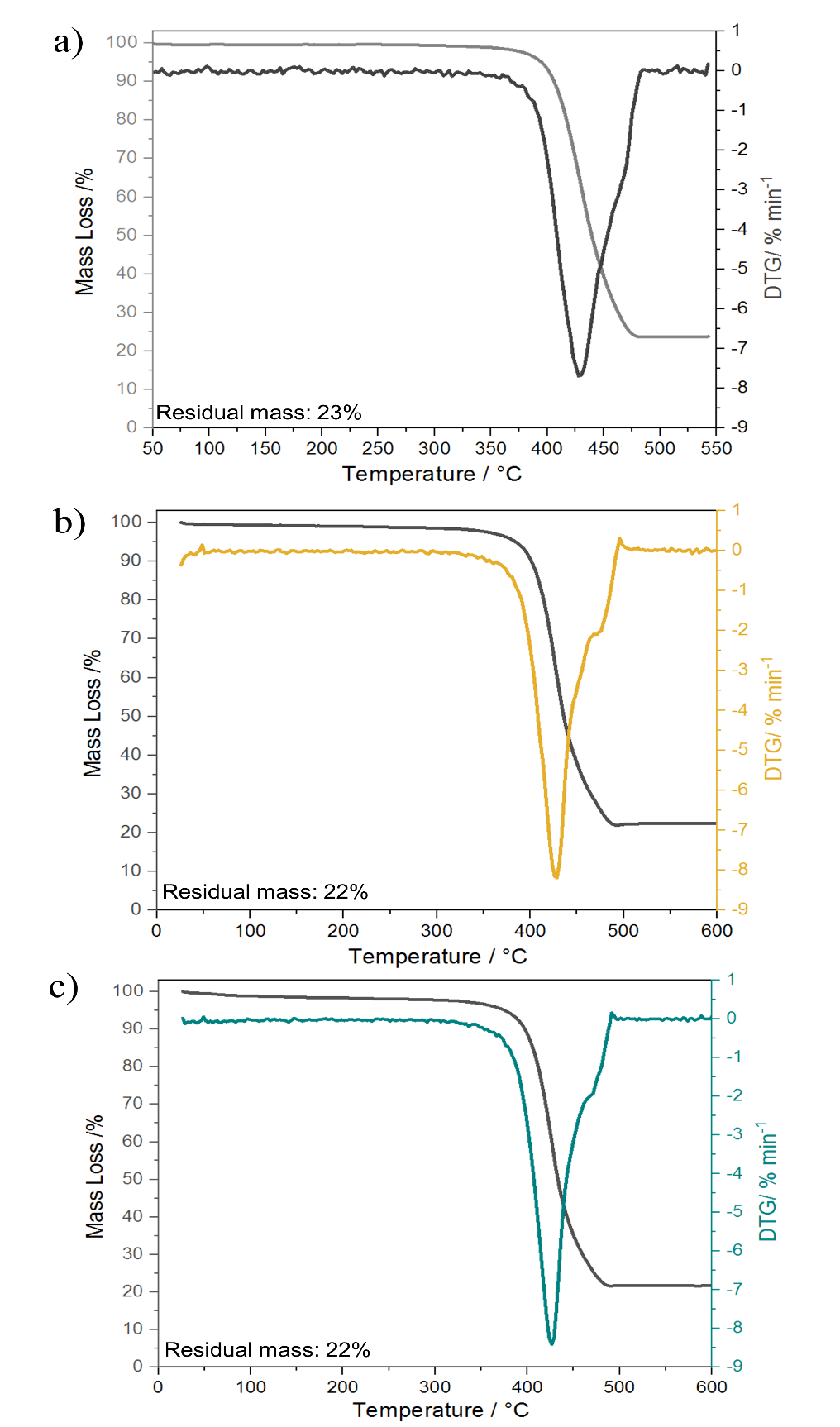


(B)

(C)

(A)

**Supplementary** **Figure 5.** TGA of sample: (A) **PAA_1**; (B) **PAA_2**; (C) **PAA_3**. The expected residual mass is 23%.

# Infrared spectroscopy (IR)


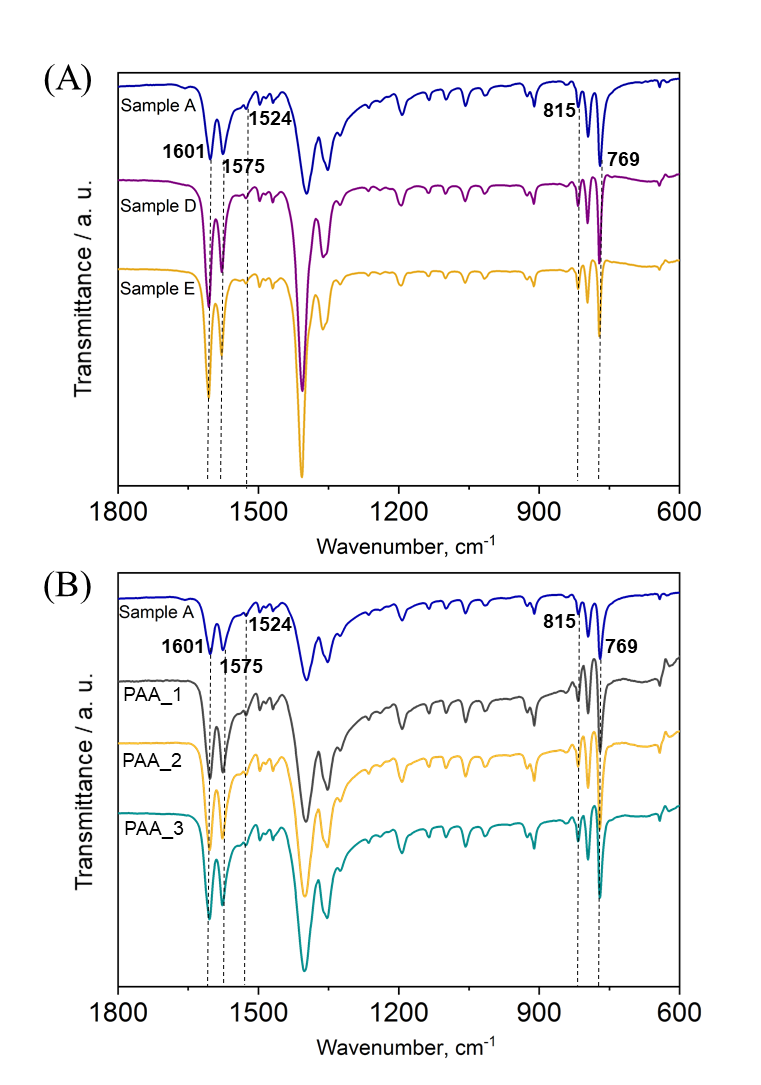


**Supplementary** **Figure 6.** FT IR spectra of: (A) samples **D** and **E** modulated by pyridine and acetic acid/pyridine; (B) samples **PAA_1**, **PAA_2**, **PAA_3** modulated by polyacrylic acid, in comparison to non-modulated sample **A**.

# Nuclear magnetic resonance spectroscopy (NMR)


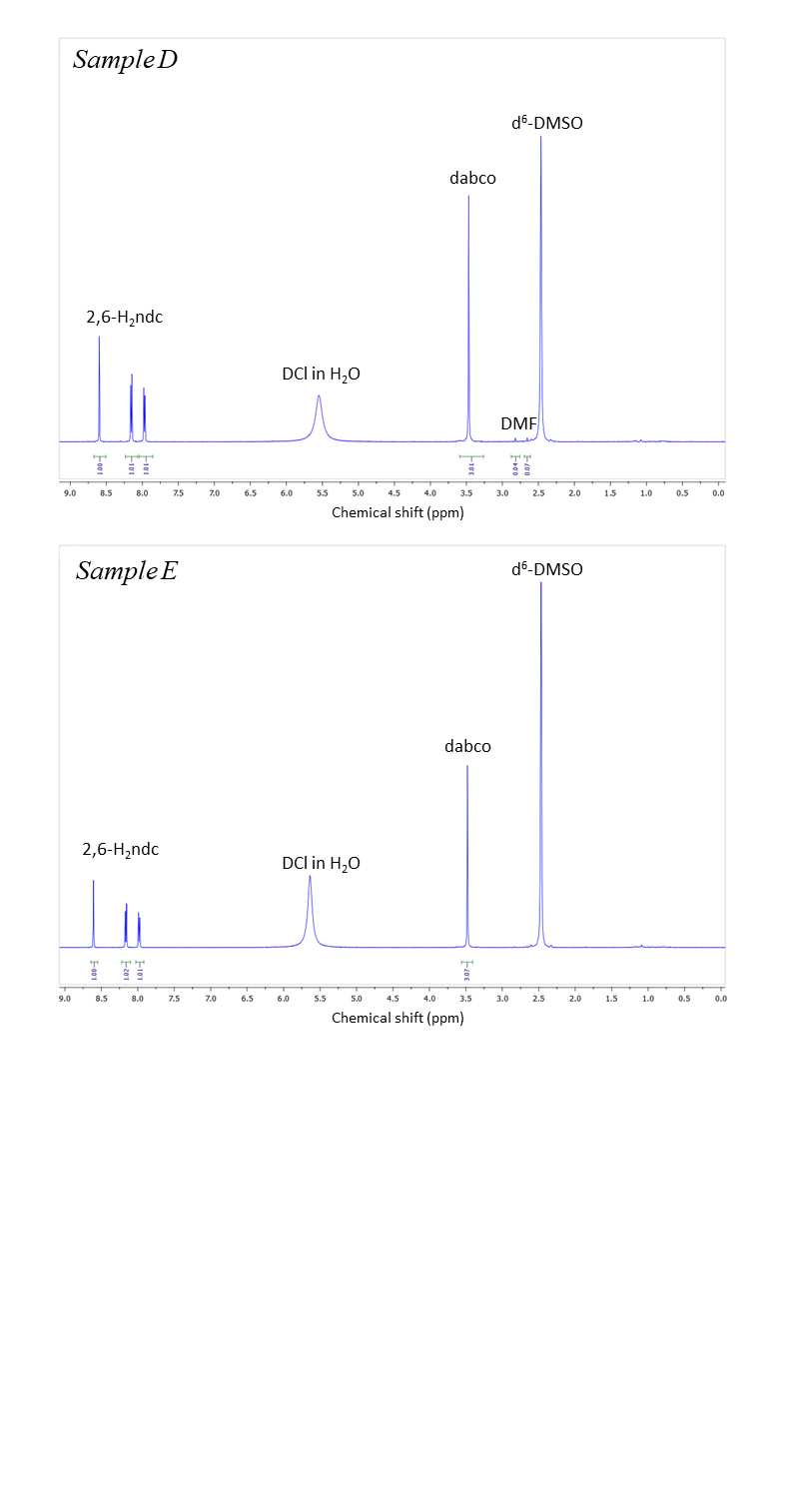


**Supplementary** **Figure 7A.** ^1^H NMR spectra of samples **D** and **E** modulated by pyridine and acetic acid/pyridine, respectively.


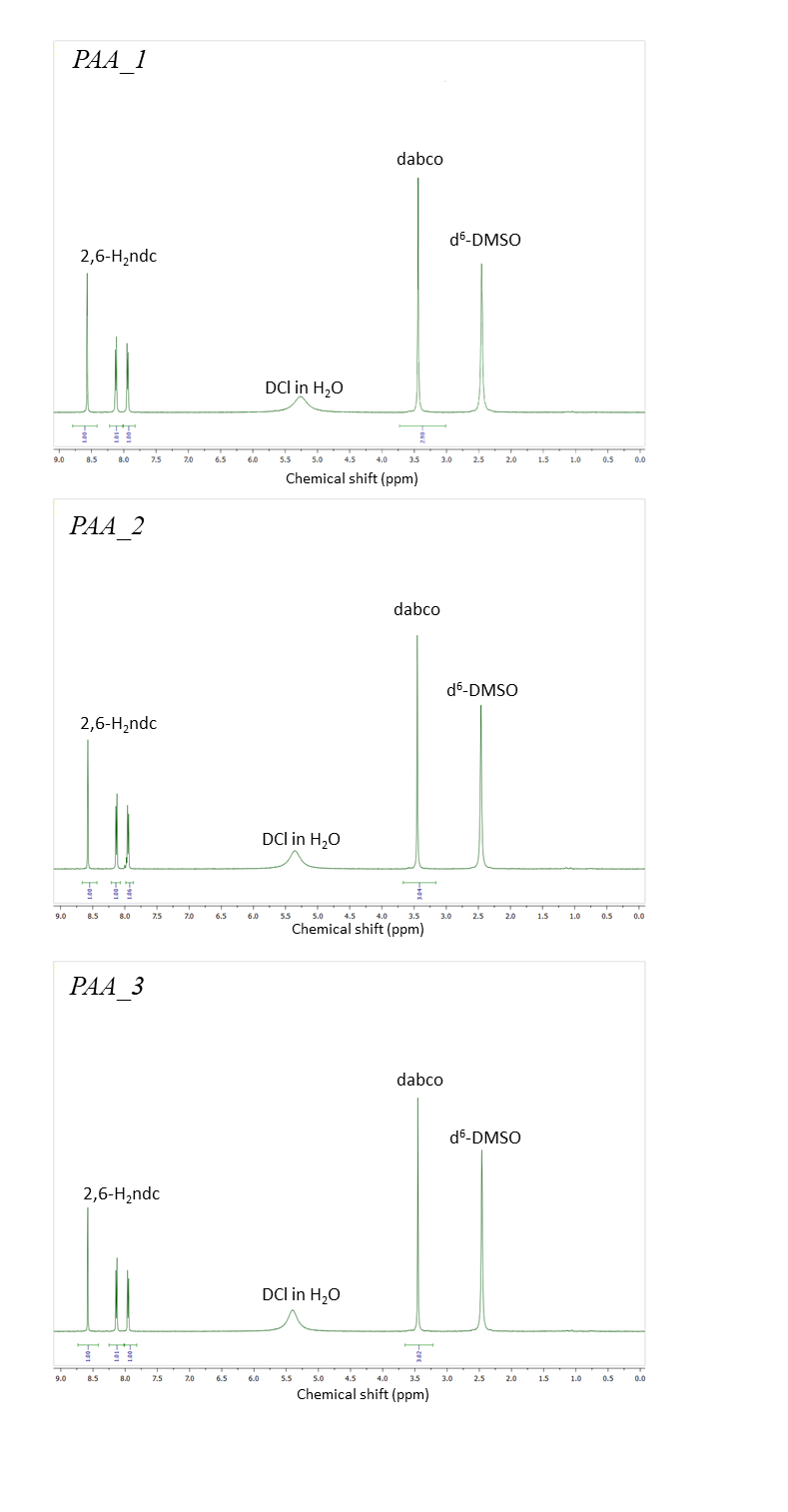


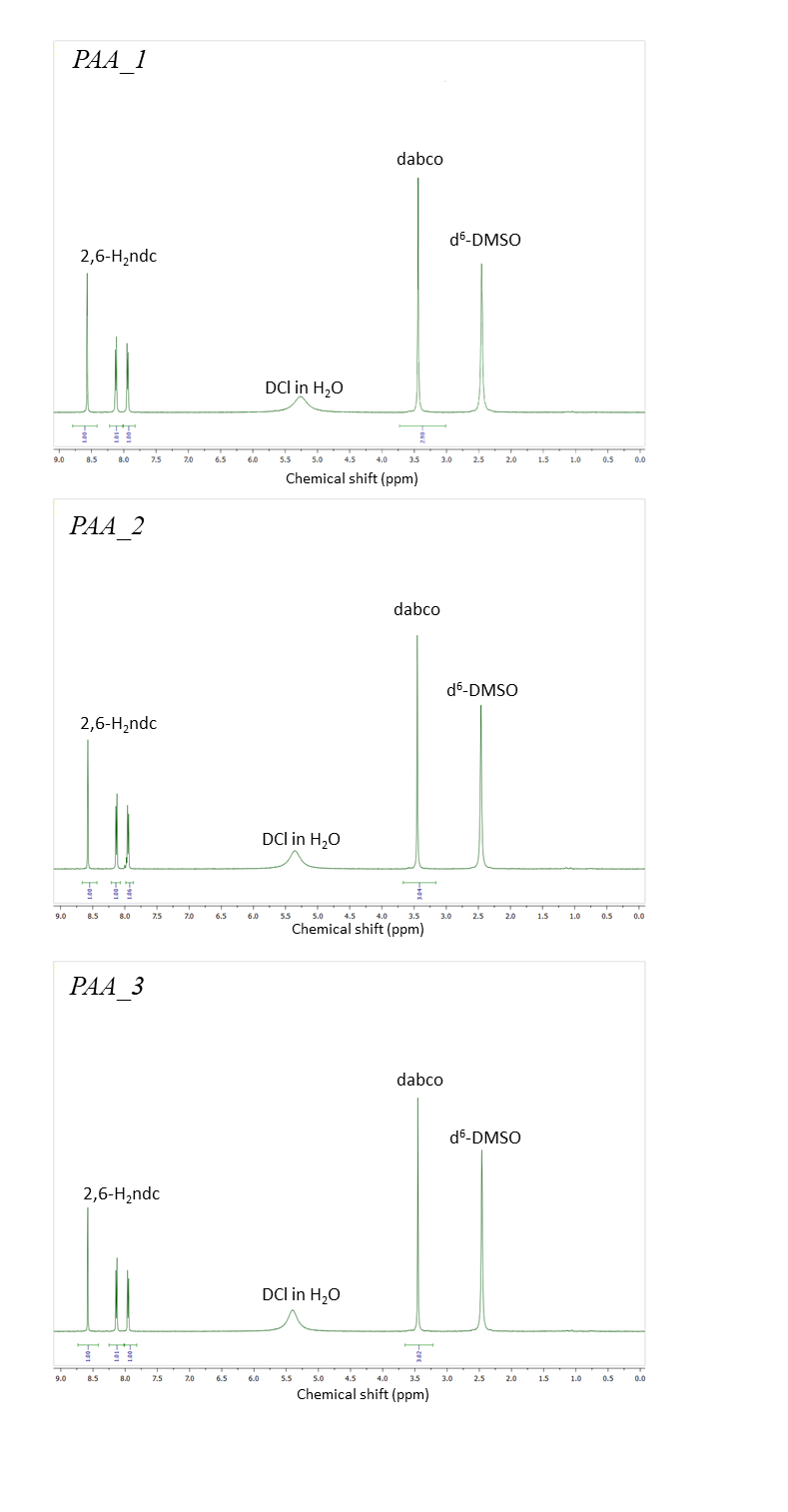


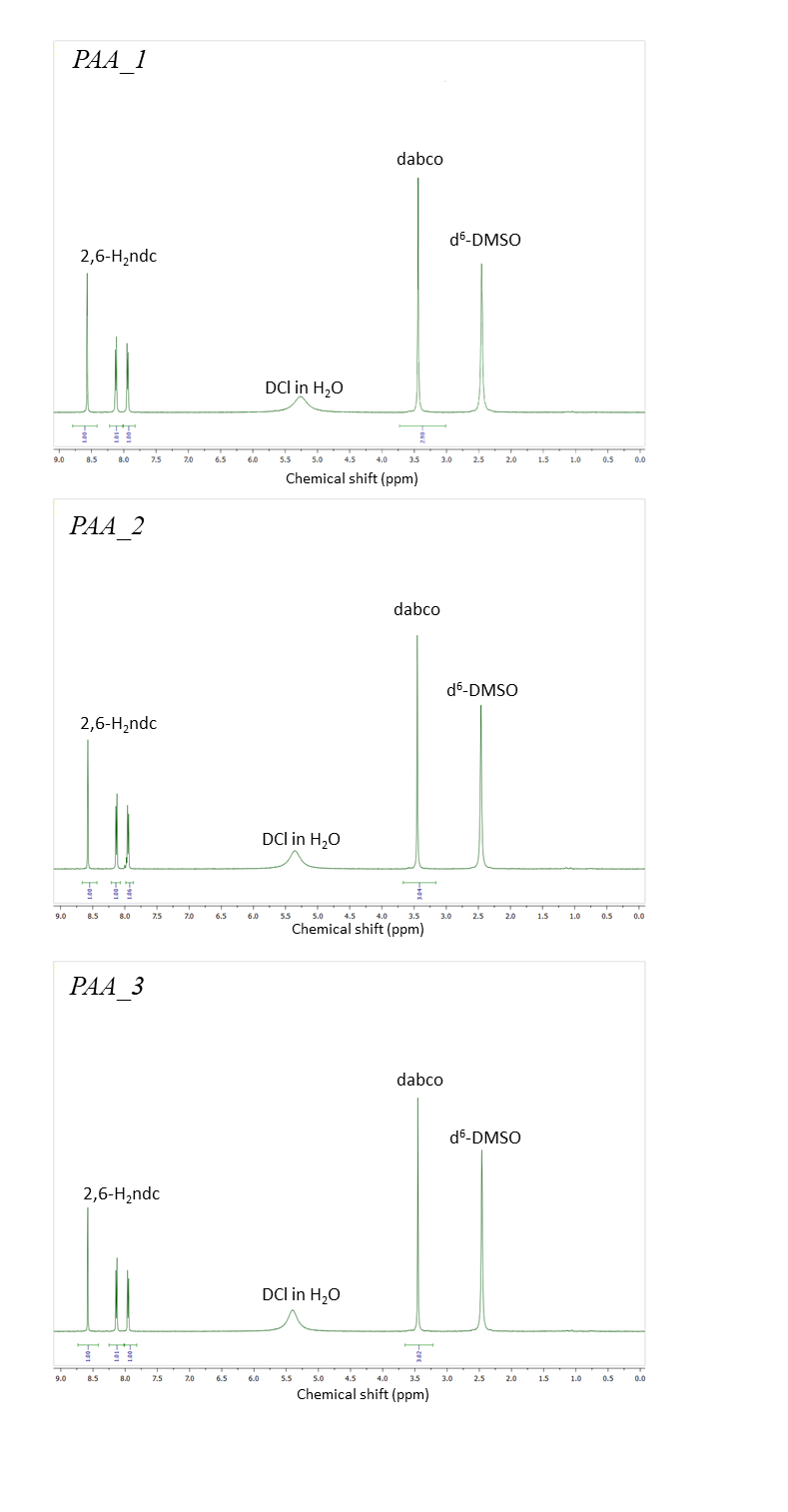


**Supplementary** **Figure 7B.** ^1^H NMR spectra of samples **PAA_1**, **PAA_2**, **PAA_3** modulated by polyacrylic acid.

# Dependence of APHM on the crystal dimensions


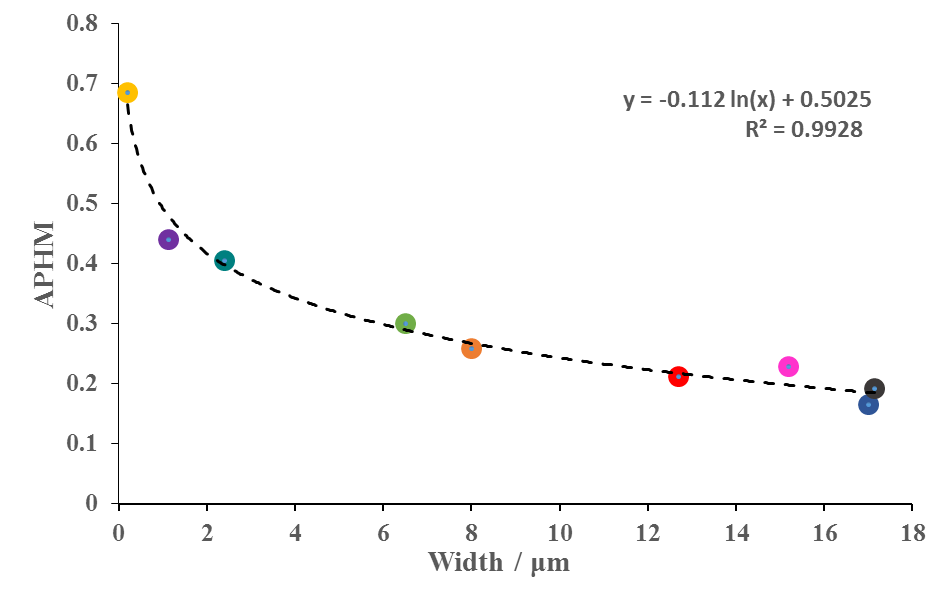


(A)

(B)


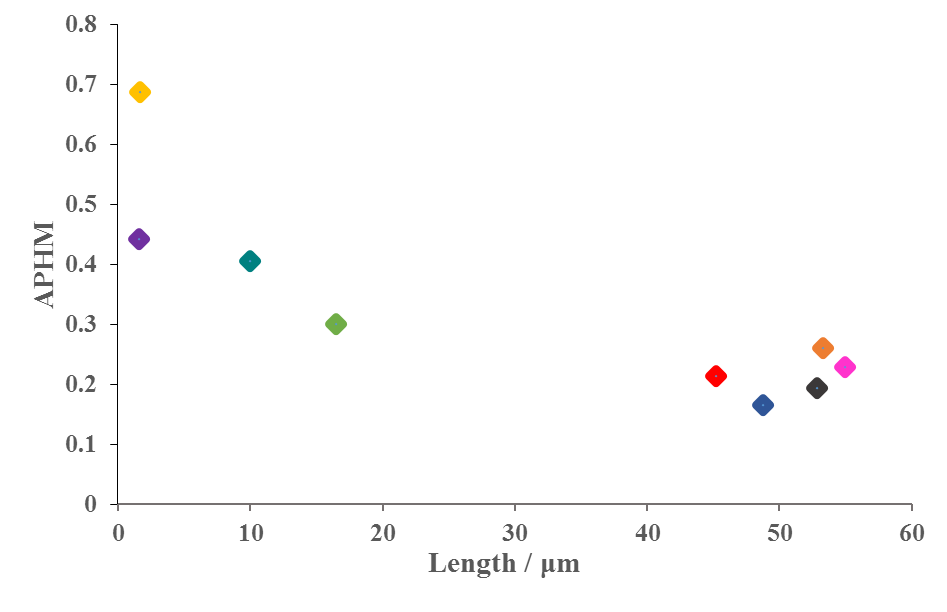


**Supplementary** **Figure 8.** Dependence of the relative adsorption pressure at half maximum uptake (APHM) from the crystal width (A) and the crystal length (B). Samples **A** – blue, **PAA-1** – black, **PAA-2** – pink, **PAA-3** – orange, **B** – red, **C** – green, sample from ref (Miura et al., 2017) – cyan, **D** – violet, **E** – yellow. The fit function is shown as a dashed line.

# Scanning electron microscopy


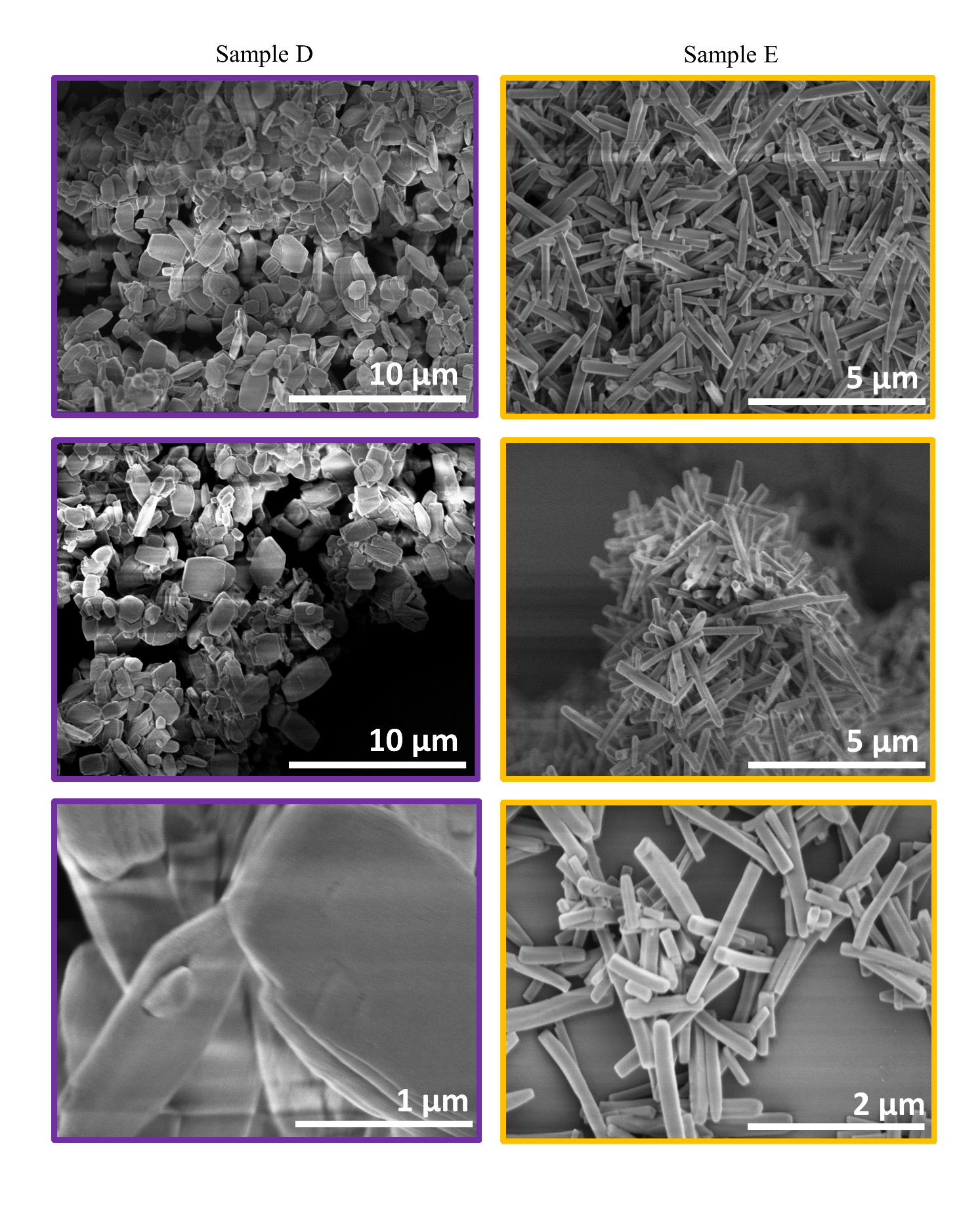
**Supplementary** **Figure 9.** SEM images of sample **D** (violet frame) and sample **E** (yellow frame).

# Analysis of the geometrical pore parameters of the static DUT-8(Ni) crystal structure


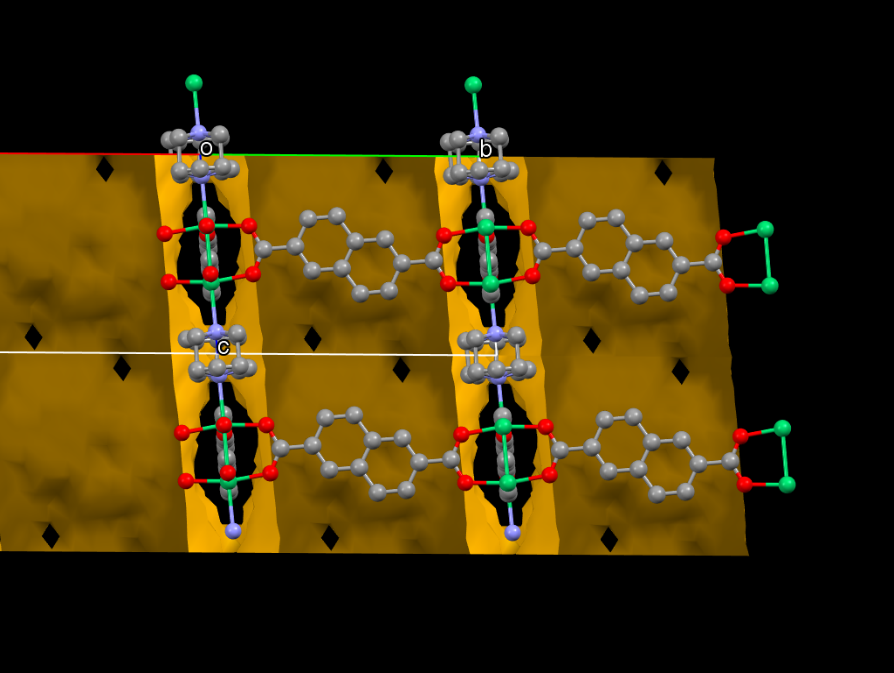


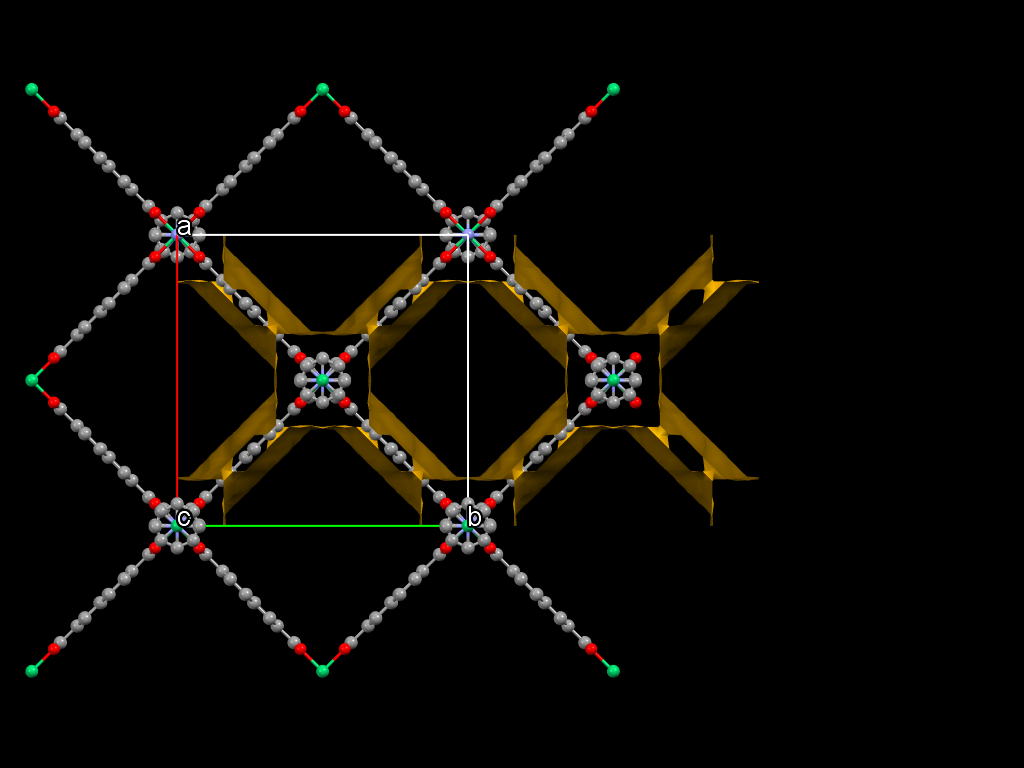


**Supplementary** **Figure 10.** View on the crystal structure along [110] (top) and [001] (bottom).

# Derivatives of adsorption isotherms


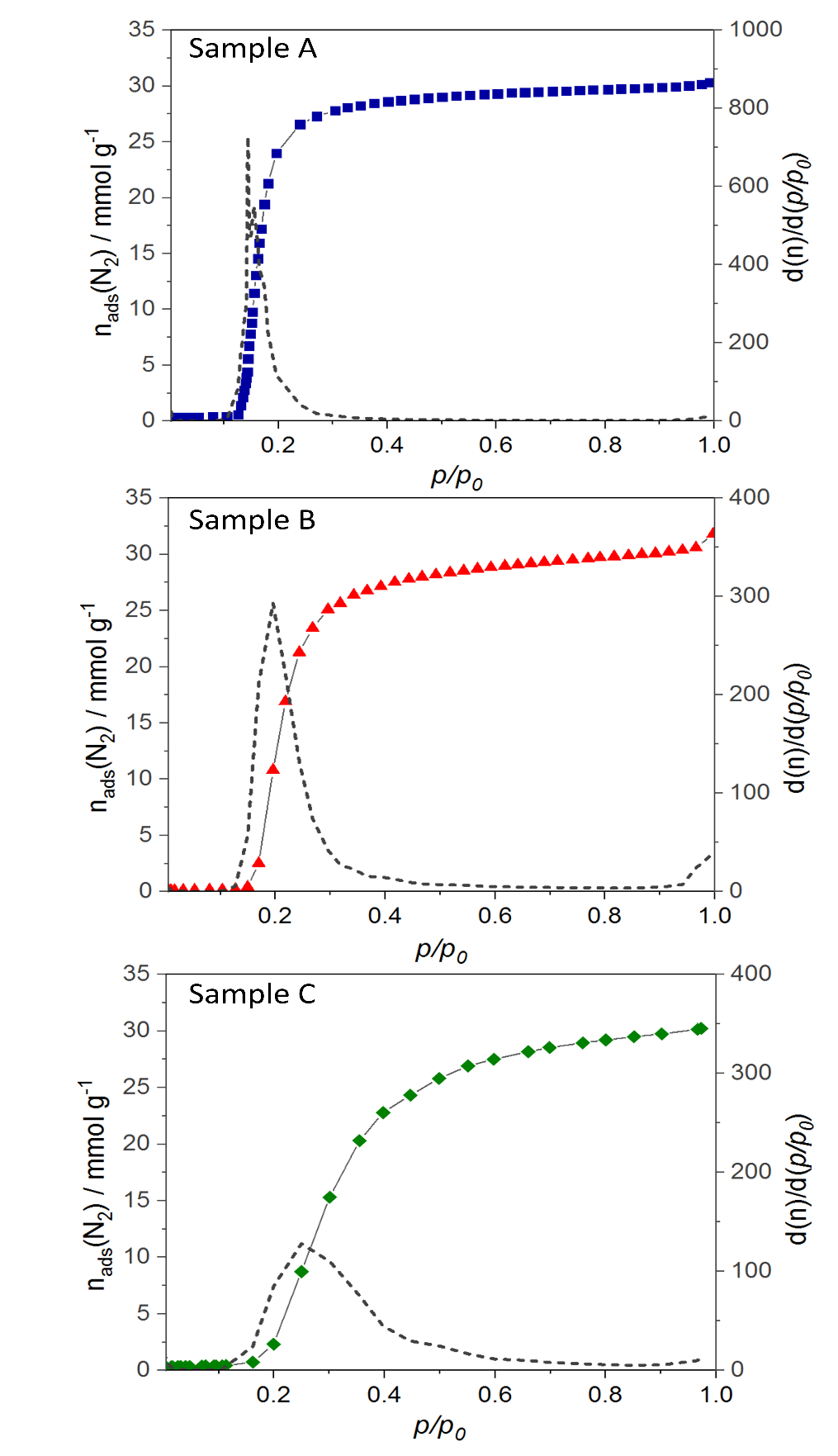


**Supplementary** **Figure 11.** The first derivatives of the adsorption branch of nitrogen physisorption isotherms at 77 K for samples **A**, **B**, and **C**.

**
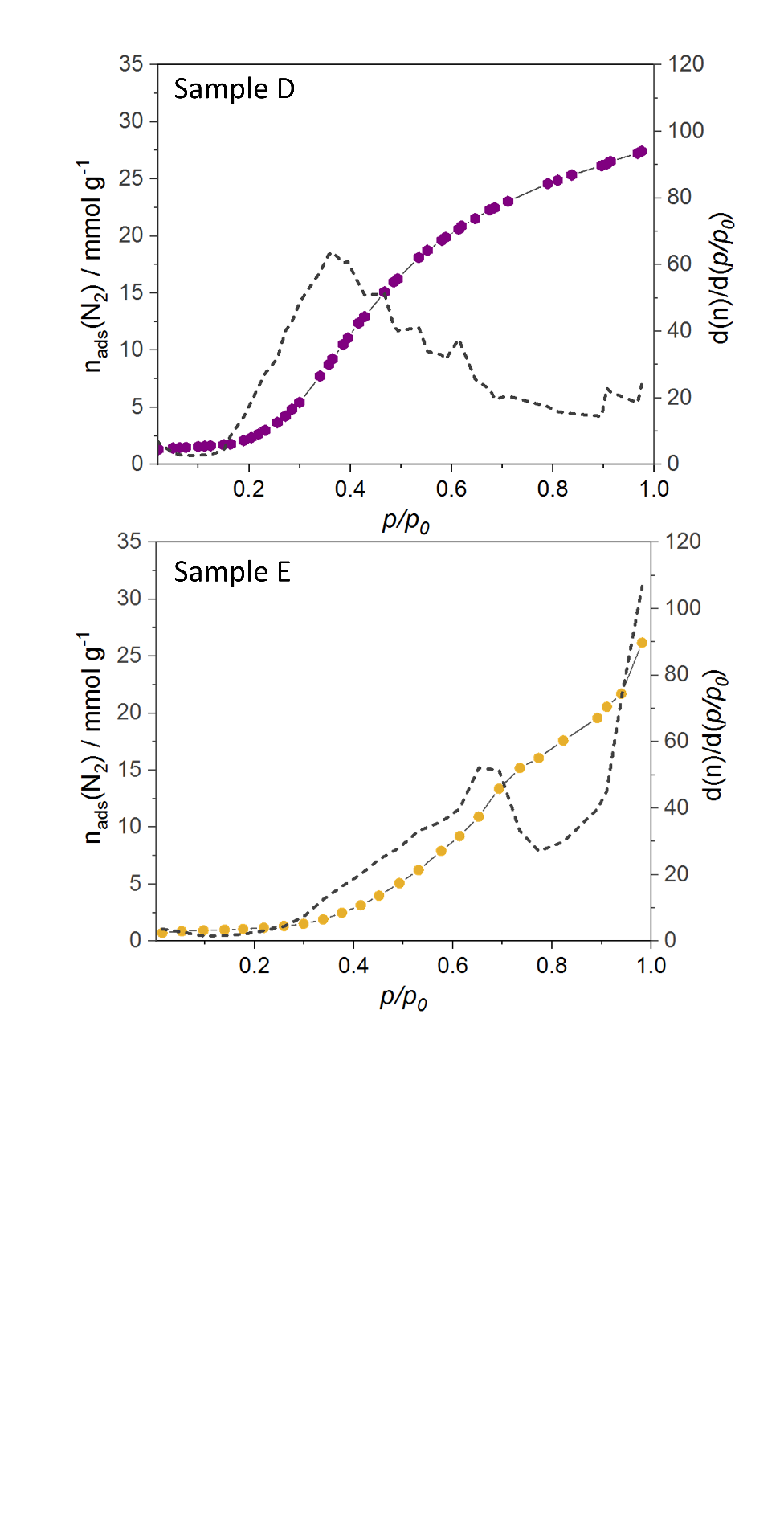
**

**Supplementary** **Figure 12.** The first derivatives of the adsorption branch of nitrogen physisorption isotherms at 77 K for samples **D** and **E**.

# Transmission electron microscopy (TEM)

**
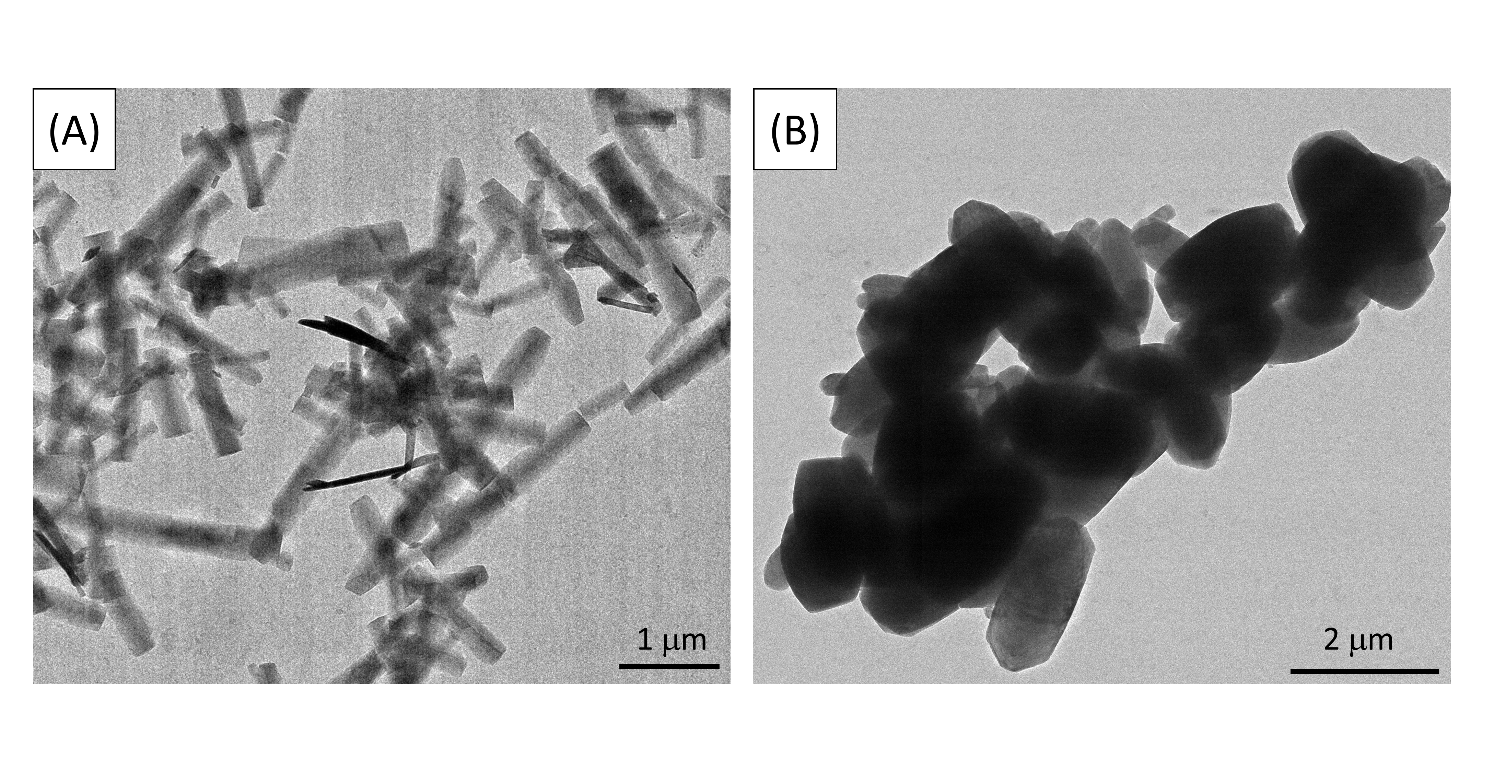
**

**Supplementary** **Figure 13.** TEM images of sample **E** (A) and sample **D** (B).

# Electron diffraction

For TEM investigations the samples dispersed in DMF were dropped onto TEM grids directly from the suspension and then dried on air.

3D electron diffraction (3D ED) data were collected using a Termofisher TITAN TEM, equipped with a 2k Gatan MSC camera, in nanodiffraction geometry with the C2 condenser aperture of 50 μm, and the effective beam diameter on the sample of 1 μm. Acceleration voltage was 300 kV. The data were collected stepwise with static pattern geometry using a dedicated Digital Micrograph (Gatan) stage controlling script (Gorelik et al., 2021). Five 3D ED datasets from individual crystals were collected for each sample. For each data set a TEM image of the crystal at zero tilt goniometer position was recorded. The total tilt rage of the goniometer was ±40° and ±50° for different datasets, depending on the position of the crystal on the TEM grid. The tilt increment was 1°, the total electron dose received by a crystal during a complete tilt series collection was 3.3e/A^2^.

Datasets were processed using EDT process software (AnaliteX, Stockholm, Sweden) supported by home-written MatLab scripts. The position of the goniometer tilt axis in imaging mode was previously determined though a tomographic series reconstruction. The azimuthal position of the tilt axis in diffraction mode was calculated in electron diffraction tomography (EDT) process.

Most of the collected datasets contained additional crystals, so that in the reconstructed reciprocal volumes obtained from 3D electron diffraction data reflections from additional crystals are present. The additional reflections influenced the unit cell determination procedure and reduced the accuracy of lattice parameter determination.

The calculated unit cell parameters:

sample **D** (Figure 14):

*a* =18.9 Å, *b*=18.6 Å, *c* =9.4 Å, *α* =90.2°, *β* =90.2°, *γ* =89.0°

sample **E** (Figure 15):

*a* = 18.5 Å, *b*=18.4 Å, *c* =9.1 Å, *α* =90.1°, *β*=91.0°, *γ*=88.4°

Both sets match well the known structure of open-pores DUT-8(Ni):

*a* =18.4312(16) Å, *c* = 9.3905(8) Å (Klein et al., 2010).

*a* = 18.576(3), *b* = 18.408(2), *c* = 9.3574(13), and *β* = 97.545(9) (Petkov et al., 2019).


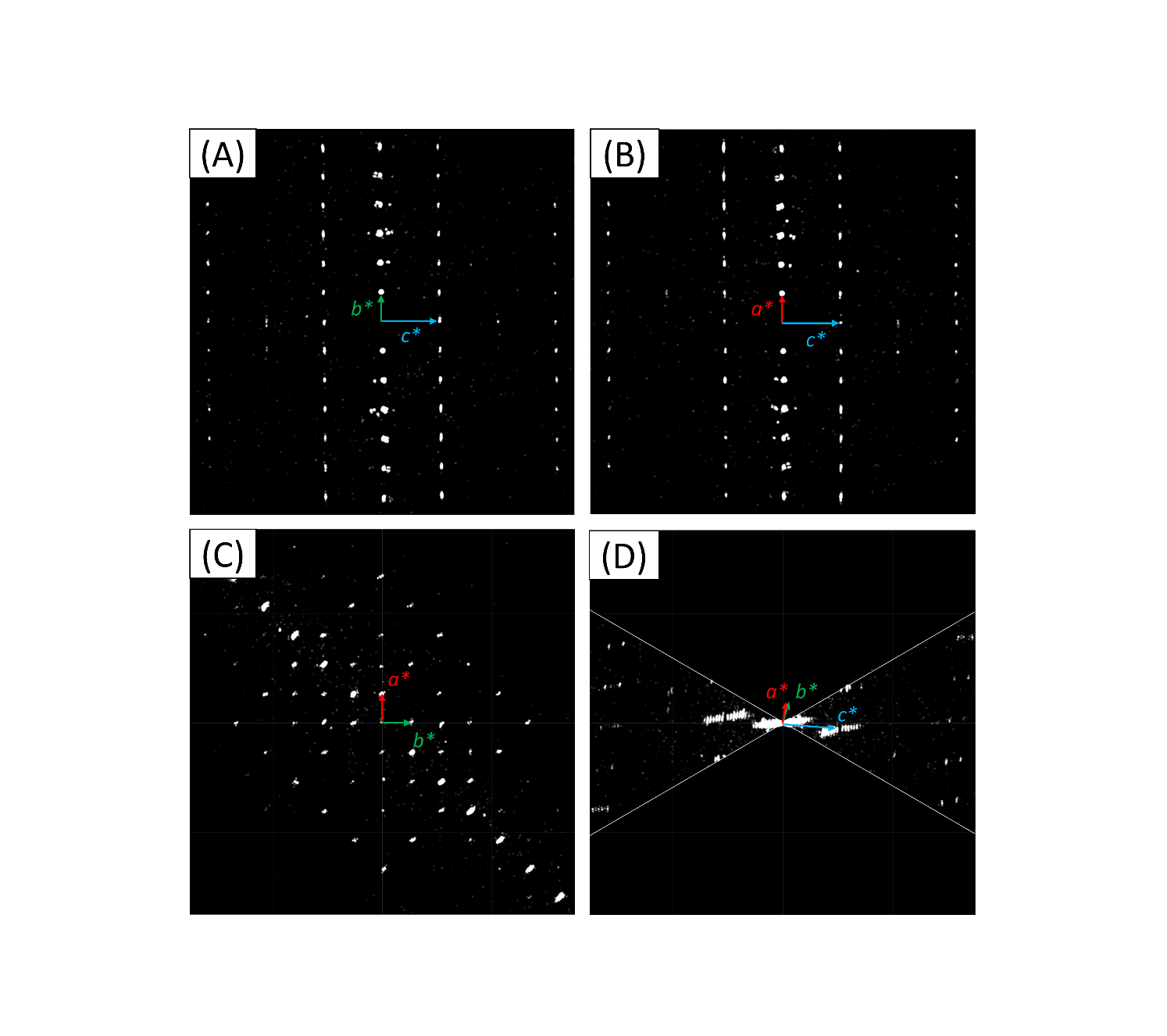


**Supplementary** **Figure 14.** Projections of reciprocal volume reconstructed from 3D ED data of sample **D**: views along *a** direction (A), *b** direction (B), *c** direction (C) and along the tilt axis of the dataset (D).

**
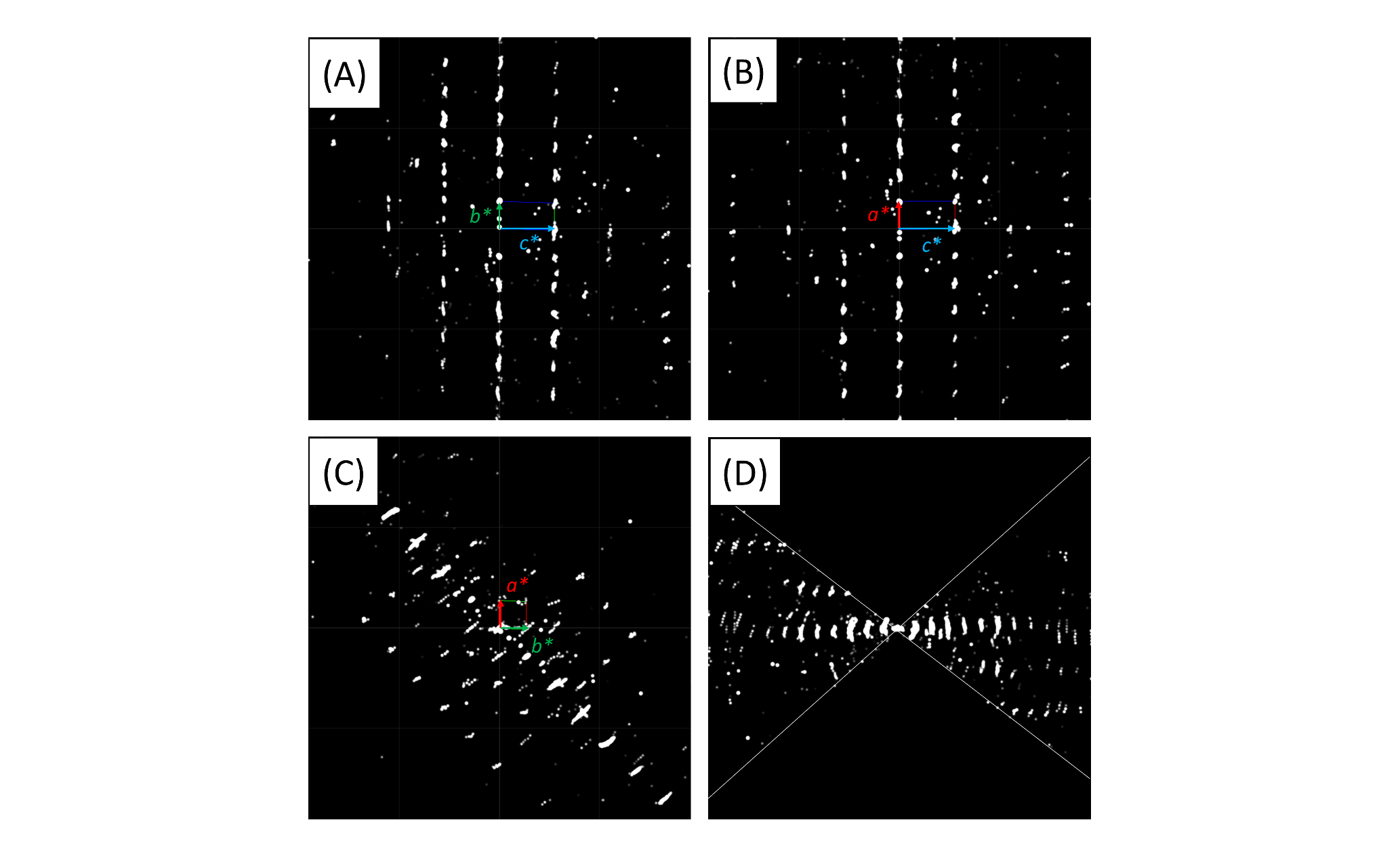
**

**Supplementary** **Figure 15.** Projections of reciprocal volume reconstructed from 3D ED data of sample **E**: views along *a** direction (A), *b** direction (B), *c** direction (C) and along the tilt axis of the dataset (D).

The obtained orientation matrices were transferred onto the corresponding images of the crystals. This procedure allowed indexing the main directions of the crystals. Figure 16 shows the orientation of the unit cell for crystals **E**. The unit cell vector *c* is running along the needle, the [110] direction coincides with the width of the crystal.


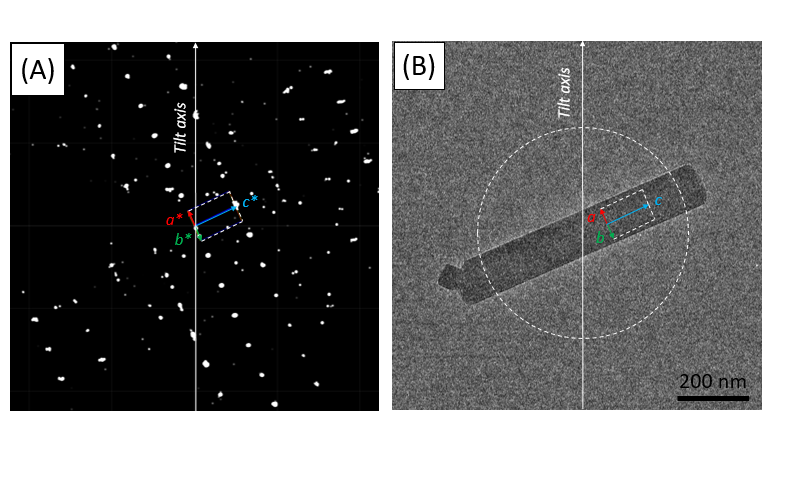


**Supplementary** **Figure 16.** Normal incidence view of the reciprocal space volume reconstructed from 3D ED data (A), showing the orientation of the reciprocal basis vectors *a*,* *b*,* and *c** (tilt axis is vertical), and corresponding image of sample **E** recorded at zero-tilt (normal incidence) with the direct space vectors *a*, *b*, *c* (B). The dashed circle represents the size of the electron beam used to collected ED data.

Figure 17 shows the orientation of the unit cell for crystals **D**. The unit cell vector *c* is running along the plate, the [110] direction coincides with the smallest dimension of the crystal.


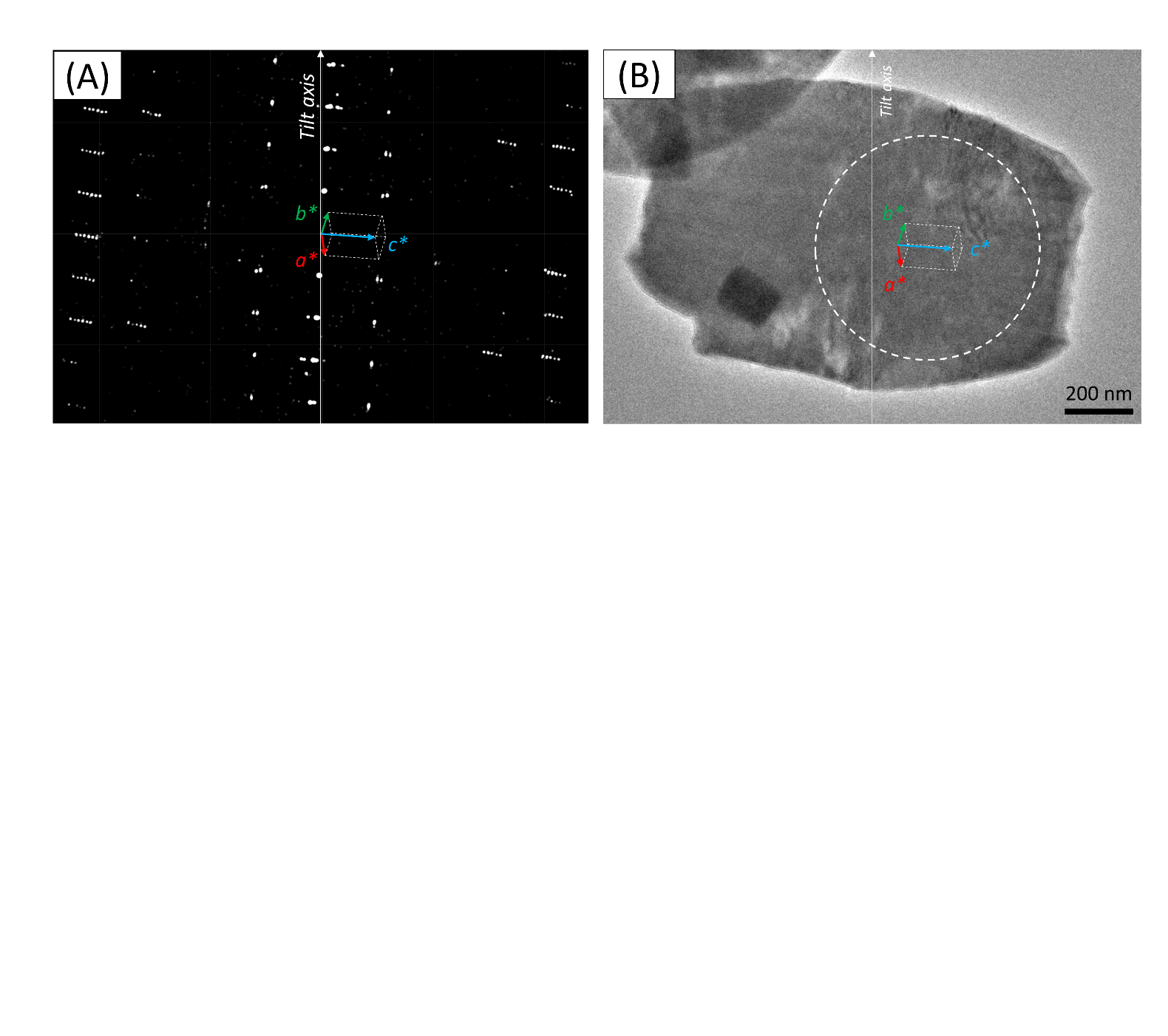


**Supplementary** **Figure 17**. Normal incidence view of the reciprocal space volume reconstructed from 3D ED data (A), showing the orientation of the reciprocal basis vectors *a**, *b**, and *c** (tilt axis is vertical), and corresponding image of sample **D** recorded at zero-tilt (normal incidence) with the direct space vectors *a*, *b*, *c* (B). The dashed circle represents the size of the electron beam used to collected ED data.

# Schematic representation of crystals dimensions


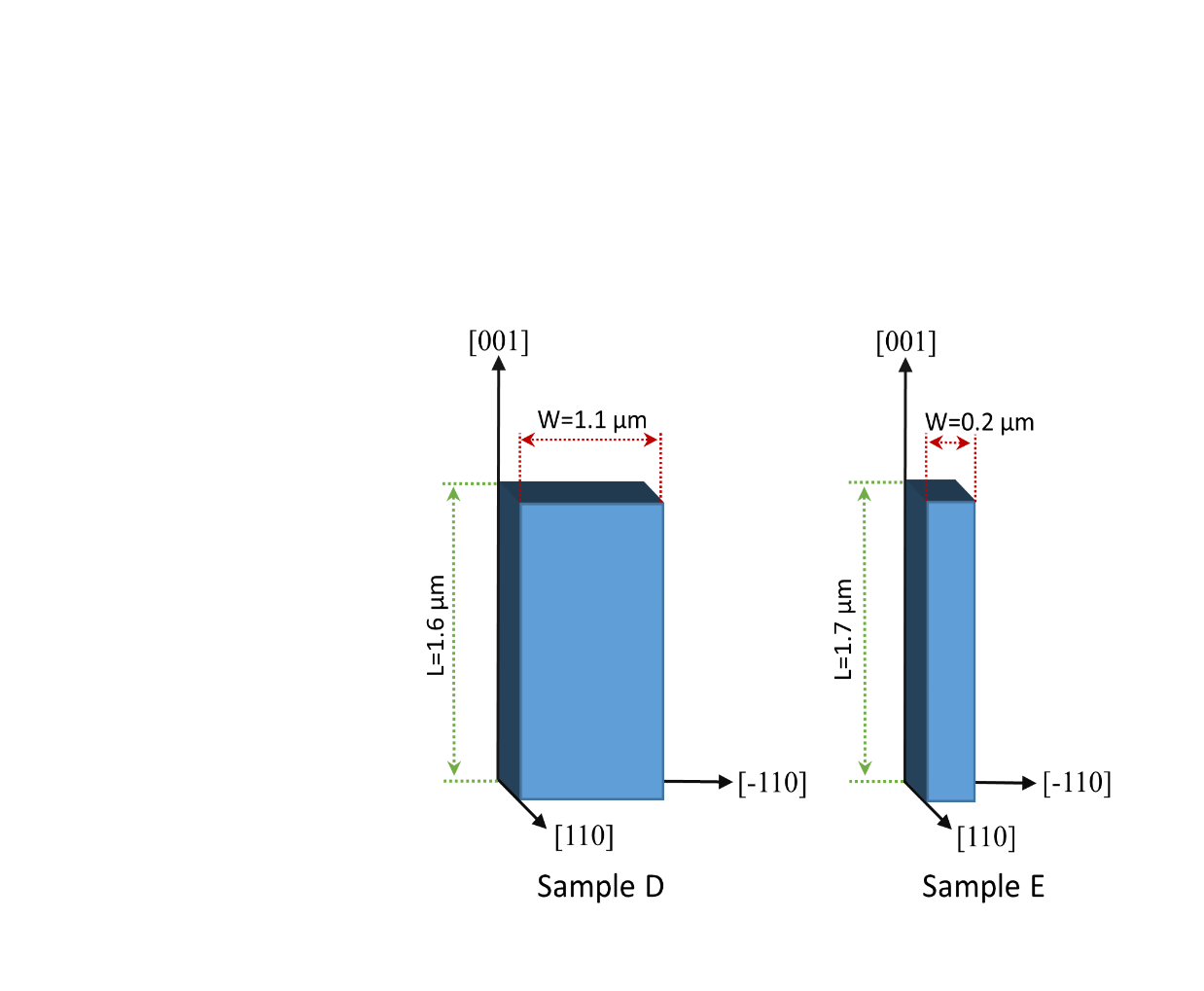


**Supplementary** **Figure 18.** Schematic comparison of lengths and widths of the plate- (sample **D**) and needle-shaped (sample **E**) crystals.

**References:**

Evans, J. D., Bon, V., Senkovska, I., Kaskel, S. (2021) A universal standard archive file for adsorption data, *Langmuir*, 37, 4222–4226. doi: 10.1021/acs.langmuir.1c00122

Gorelik, T. E., Nergis, B., Schöner, T., Köster, J., Ute Kaiser, U. (2021). 3D electron diffraction of mono- and few-layer MoS_2_. *Micron* 146, 103071. doi: /10.1016/j.micron.2021.103071.

Miura, H., Bon, V., Senkovska, I., Ehrling, S., Watanabe, S., Ohba, M., et al. (2017). Tuning the gate-opening pressure and particle size distribution of the switchable metal–organic framework DUT-8(Ni) by controlled nucleation in a micromixer. *Dalton Trans.* 46, 14002 14011. doi: 10.1039/C7DT02809A

Klein, N., Herzog, C., Sabo, M., Senkovska, I., Getzschmann, J., Paasch, S., et al. (2010). *Phys. Chem. Chem. Phys*. 12, 11778-11784. [doi: 10.1039/C003835K](https://doi.org/10.1039/C003835K)

Petkov, P. S., Bon, V., Hobday, C. L., Kuc, A. B., Melix, P., Kaskel, S., et al. (2019). Conformational isomerism controls collective flexibility in metal–organic framework DUT-8(Ni). *Phys. Chem. Chem. Phys.* 21, 674–680. doi: 10.1039/C8CP06600K
